# Modeling and Experimental Methods to Probe the Link between Global Transcription and Spatial Organization of Chromosomes

**DOI:** 10.1371/journal.pone.0046628

**Published:** 2012-10-01

**Authors:** K. Venkatesan Iyer, Shovamayee Maharana, Soumya Gupta, Albert Libchaber, Tsvi Tlusty, G. V. Shivashankar

**Affiliations:** 1 National Centre for Biological Sciences, Tata Institute for Fundamental Research, Bangalore, Karnataka, India; 2 Laboratory of Experimental Condensed Matter Physics, Rockefeller University, New York City, New York, United States of America; 3 Department of Physics of Complex Systems, Weizmann Institute of Science, Rehovot, Israel; 4 Mechanobiology Institute and Department of Biological Sciences, National University of Singapore, Singapore, Singapore; University of Maryland School of Medicine, United States of America

## Abstract

Genomes are spatially assembled into chromosome territories (CT) within the nucleus of living cells. Recent evidences have suggested associations between three-dimensional organization of CTs and the active gene clusters within neighboring CTs. These gene clusters are part of signaling networks sharing similar transcription factor or other downstream transcription machineries. Hence, presence of such gene clusters of active signaling networks in a cell type may regulate the spatial organization of chromosomes in the nucleus. However, given the probabilistic nature of chromosome positions and complex transcription factor networks (TFNs), quantitative methods to establish their correlation is lacking. In this paper, we use chromosome positions and gene expression profiles in interphase fibroblasts and describe methods to capture the correspondence between their spatial position and expression. In addition, numerical simulations designed to incorporate the interacting TFNs, reveal that the chromosome positions are also optimized for the activity of these networks. These methods were validated for specific chromosome pairs mapped in two distinct transcriptional states of T-Cells (naïve and activated). Taken together, our methods highlight the functional coupling between topology of chromosomes and their respective gene expression patterns.

## Introduction

The genetic material (chromatin) in eukaryotic cells has a multi-scale three dimensional organization within the nucleus [1]. DNA is packaged around histone and non-histone proteins to form the 30 nm chromatin fibre [2]. This 30 nm fibre is further hypothesized to be organized into relatively open euchromatin and condensed heterochromatin structures based on post translational modifications of histone [3]. Imaging methods using whole chromosome probes (FISH) reveal the spatial dimension to genome organization in eukaryotic cells. These methods have suggested that chromatin is organized into well-defined chromosome territories (CT), in a tissue specific non-random manner [4]–[7]. These chromosome positions remain largely conserved during the interphase in proliferating cells [8]–[10]. In addition, whole genome chromosome conformation capture assays have shown intermingling of neighbouring CTs [11] as well as a model of the yeast genome organization [12]. Further on a smaller scale, these methods have demonstrated that the genes from neighbouring CTs loop out and are found to co-cluster with transcription machinery to form three dimensional interactions called active transcription hubs [13]. The intermingling of nearby CTs vary in concert with transcription and cellular differentiation [14], [15], demonstrating the role of chromosome topology in genome regulation [16]. Individual gene labeling methods suggest that candidate gene clusters are spatially co-localized [17] and are co-regulated for their specific transcriptional control [18]–[24]. Using 2D matrices of chromosome distances at prometaphase stage, the correspondence between co-regulated genes and chromosome positioning has been observed during differentiation [19]. However, methods to describe the correlations between three-dimensional architecture of chromosome positions [25], [26] and global gene expression as well as TFNs is largely unexplored.

In this paper, we present a quantitative approach to test the correlation between chromosome organization and transcriptional output of the cell. Inter-chromosome Physical Distance (IPD) matrix computed from chromosome centroids in interphase human male fibroblasts [27] revealed non random chromosome organization. Inter-chromosome Activity Distance matrix, constructed from the microarray data obtained for human fibroblast [28], suggested that chromosomes with similar gene activity were spatially clustered in a tissue specific manner. We formulate an energy optimization function, ‘H’ to elucidate the correspondence between the annotated TFNs [29] and spatial positioning of chromosomes. Numerical simulations of the H function, that relates the activity of genes of specific networks to their corresponding chromosomal positions, suggest the sensitivity in network topology. The prediction from our numerical methods were experimentally validated by correlating chromosome distances for specific pairs with their respective activity distances in two distinct transcriptional states of murine T-Cells (naïve and activated). Taken together these numerical modeling and experimental methods provide an important platform to probe the functional coupling between spatial organization of chromosomes and their epigenetic states.

## Results

### Methods to probe the correlation between the organization of chromosomes and their transcriptional activity

**Figure 1:**
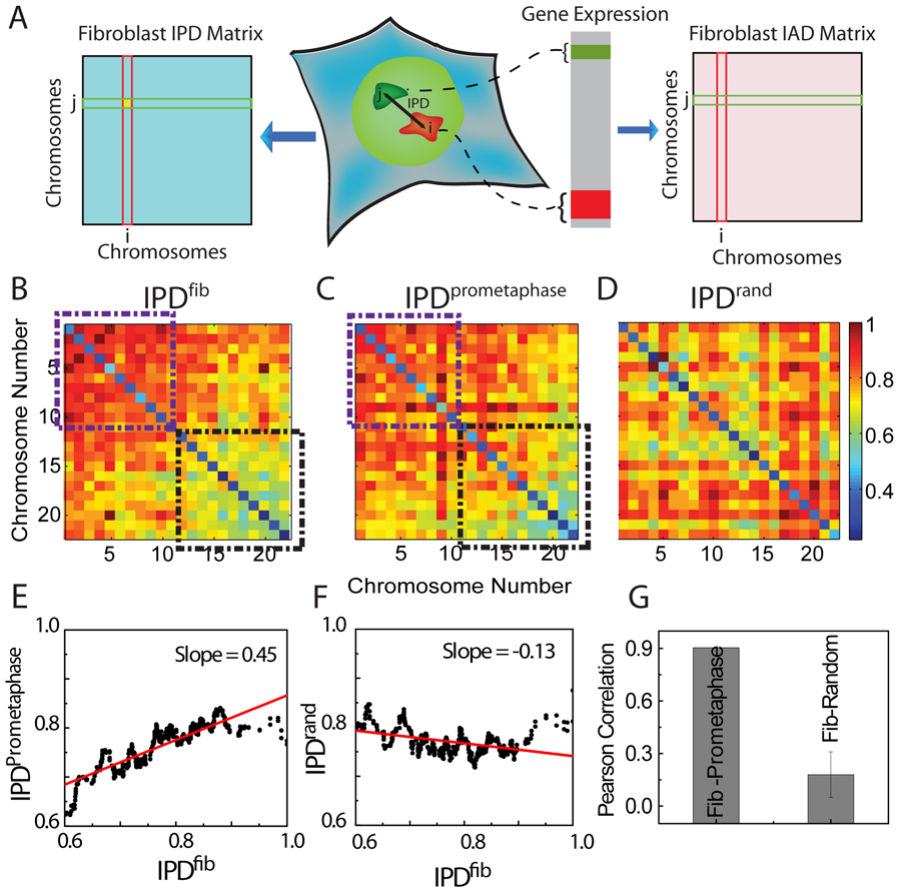
Generation of IPD matrices. (**A**) Schematic description of the procedure for generating the IPD^fib^ matrix from the mean distances between pairs of chromosome centroids in the nuclei of human fibroblasts, and the IAD^fib^ matrix from microarray expression data grouped into chromosomes. (**B**) IPD^fib^ matrix for interphase fibroblasts represented in color code: warmer colors represent larger inter-chromosome distances and cooler colors smaller distances. Rows and columns indicate chromosome number. (**C**) IPD^prometaphase^ matrix for prometaphase stage of fibroblasts. Black and violet boxes in (B) and (C) represents clusters of chromosome pairs showing smaller and larger IPD values respectively.(**D**) IPD^rand^ matrix for randomized chromosome positions obtained by randomizing IPD^fib^(Color scale bar-Inter-chromsome distance, in units of fraction of nuclear radius). (**E**) Scatter plot between IPD^fib^ and IPD^metaphase^. (**F**) Scatter plot between IPD^fib^ and IPD^rand^. (**G**) Pearson correlation coefficients for correlations in (E) and (F). In case of IPD^rand^, the PCC was the mean of hundred randomizations.



3D Chromosome FISH was used to map chromosome positions in two cell phases: interphase and prometaphase [27], [30]. Based on these observations we extracted the coordinates of all chromosome centroids in human fibroblasts measured for 54 nuclei, as reported by Bolzer *et al.* [27], which is the only available full map of all chromosome positions. Inter-chromosome Physical Distance (IPD) matrices were constructed by the mean distances between centroid positions of 22 pairs of autosomes (Figure 1A) as:

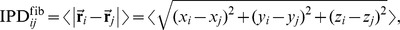
where **r_i_ = **(*x_i_, y_i_*, *z_i_*) and **r_j_ = **(*x_j_, y_j_*, *z_j_*) are the coordinates of chromosome *i* and chromosome *j*, respectively and < > denotes averaging over the 54 nuclei. Figure 1A shows Inter-chromsome physical distance between ith and jth chromosome in the nucleus, which represents the (i,j)^th^ element in the IPD matrix. The IPD matrices were constructed for interphase (IPD^fib^, Figure 1B), prometaphase (IPD^prometaphase^, Figure 1C) and randomized nucleus (IPD^rand^, Figure 1D). The values of diagonal elements of all the matrices, which represent mean distance between homologues, are kept minimal and are not considered for any further correlation analysis. Further, regions of low IPD values and high IPD values are observed in the IPD matrices for interphase and prometaphase (Figure 1B and 1C), suggesting contribution of chromosome size (in total number of base pairs) which decreases from chromosome 1 to 22 (Figure S1A). The volume of a given chromosome changes dramatically in interphase [31] due to changes in epigenetic modification and subsequent transcriptional states. Hence IPD is an average of chromosome centroids in all such conditions showing spatial clustering of chromosomes. Such clustering was not observed in the randomized nucleus, where these matrices were generated by randomly swapping the rows and columns of IPD matrix multiple times and hence permuting the identities of the chromosomes in the interphase IPD matrix (Methods). Pearson Correlation Coefficient (PCC) estimation between initial IPD and progressive randomization showed significant decrease in PCC values after 30 such permutations (Figure S2). Interphase and prometaphase IPDs were found to be positively correlated (Figure 1E) with PCC of 0.904 (Figure 1G), whereas IPD^fib^ was uncorrelated with the randomized position matrix (mean PCC of 0.17 with standard deviation of 0.13, computed over 10,000 randomized matrices) (Figure 1F - a representative scatter plot, 1G and Figure S3), confirming the non-random organization of the chromosomes in interphase cell nucleus.

**Figure 2:**
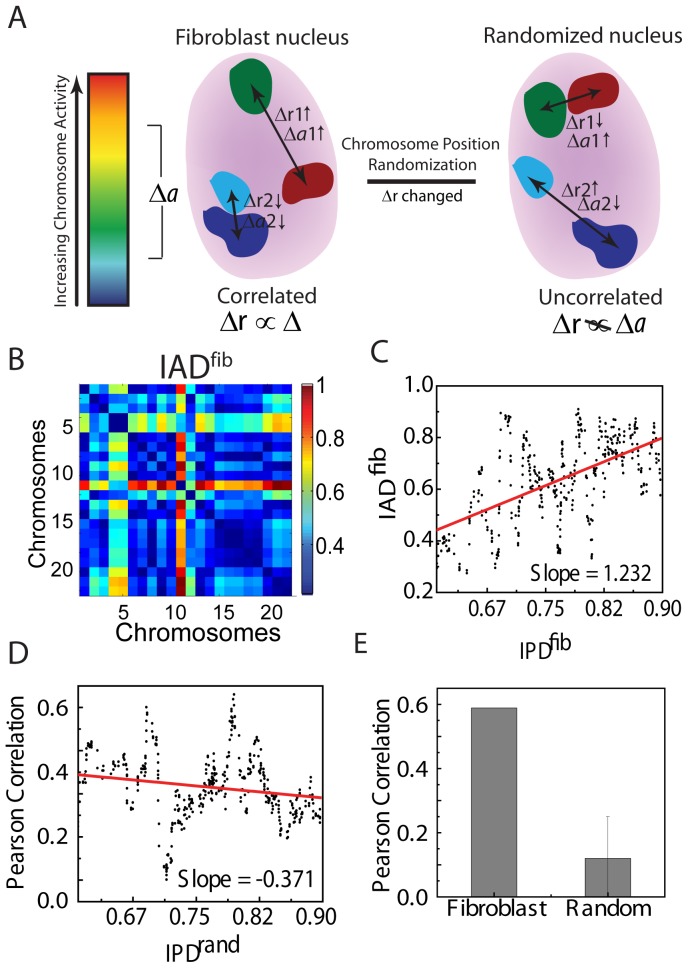
Generation of IAD matrices and its correlations with IPD. (**A**) Schematic representation of the hypothesis that position of chromosomes is correlated to the activities of the chromosomes. (**B**) IAD^fib^ matrix for interphase fibroblast, in arbitrary units. Warmer color shows larger difference in activities and cooler color indicates smaller differences. (**C**) Scatter plot between IPD^fib^ and IAD^fib^. (**D**) Scatter plot between IPD^rand^ and IAD^fib^. (**E**) Pearson correlation coefficient for the correlations in (D) and (E).



To probe the possible correlation between the chromosome positions and their gene activity (Figure 2A), we generated an Inter-chromosomal Activity Distance (IAD) matrix for fibroblast from the microarray data (Figure 2B) obtained from Goetze *et al* [28]. Figure 1A shows the schematic of gene activities being classified to i^th^ and j^th^ chromosomes, which is then further used to compute the (i,j)^th^ value in the IAD matrix. From the microarray data, genes were grouped into their respective chromosomes and the mean logarithmic chromosomal activities (*A*chr) were obtained (Methods). The density of genes on a chromosome does not correlate with the length of the chromosomes (Figure S1A). For instance, chromosome 18 is larger than chromosome 19, but the former has smaller number of genes as compared to the latter (Figure S1B). Considering this, chromosomal activity was computed by normalizing total activity of all the genes by the annotated number of genes and not by the chromosome size. Interchromosome Activity Distance (IAD) was then computed as:

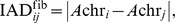



Use of logarithmic scale captures expression levels over several orders of magnitude. Lower IAD values, shown by cooler colors in the heat map, represent - pairs with similar transcriptional activity, whereas warmer colors represent higher IAD or dissimilar chromosomal activities, as seen in Figure 2B. The correlations between IAD^fib^ and IPD^fib^ matrices at interphase (Figure 2C), revealed a positive slope (1.23) and PCC (0.58, Figure 2E) with a small false discovery rate (FDR) 0f 0.11 (Figure S4 and Table S1). To probe the specificity of this correlation, we used the IPD at prometaphase (IPD^prometaphase^) as a negative control. Indeed, we obtained a lower slope (0.44) and PCC (0.27) when IAD^fib^ (for interphase) was correlated with IPD^prometaphase^ (Figure S3 B), with a larger FDR ∼0.29 (Table S1 & Figure S4C) suggesting that IPD at interphase is more correlated with IAD at interphase. Correlation with randomized matrix exhibited negative slope (−0.37, Figure 2D) for a typical randomized matrix (Figure 1D) and even smaller average PCC of 0.12 (Figure 2E & Figure S3D) further indicating the non-randomness in the correlation. To probe the effect of chromosome size on the observed correlation between IPD and IAD, we generated a matrix of chromosome basepair length differences (Interchromosome Basepair Distance, IBD) (Methods and Figure S1C). This matrix showed some degree of similarity to IPD and correlated well with the IPD matrix (PCC ∼0.54) (Figure S1D & S1F). But when IBD was correlated with the IAD matrix, a very weak correlation of PCC ∼0.15 was observed (Figure S1E and S1F). This suggested that though chromosome size contribute to the observed pattern in IPD, the correlation between IPD and IBD were not influenced by the chromosome sizes. The Interchromosomal Activity Distance matrix used in the correlations was generated by computing the mean of the genes present in the chromosome. This takes into account all the genes irrespective of their activity level. To probe the correlation due to a small subset of genes on the chromosome, which will correspond to smaller active regions of chromosomes (as the active genes are not uniformly distributed throughout the length of chromosome), we selected genes (∼25% of the genome) which are highly expressing in each chromosome by applying a threshold (more than 40% of the mean chromosome activity) in the gene expression. We generated the IAD matrix (Figure S5A) from these selected genes (IAD^select^) and calculated the correlations. The correlation obtained after selection of genes was similar to the correlation when all genes in the chromosome were used (Figure S5B & S5C), suggesting that the correlation is not due to whole chromosome averaging. These results suggests that the mean distances between chromosomes are more correlated with gene activity distances in fibroblasts at interphase as compared to prometaphase and are uncorrelated with random organization.

### Methods to identify cell-type specific gene expression profiles and its correlation to chromosome positions

Different cell types in an organism are characterized by their distinct transcriptomes. Correlation of gene expression to chromosome organization implies that cell types will differ in the positions of the chromosomes, such that the spatial organization of a given cell type exhibits larger correlation with its own expression pattern. In the presence of cell type specific correlation between IPD and IAD, the correlation should be smaller when IPD^fib^ is correlated with IAD of other cell types. To further extend our approach to test such cell type specific correspondence, we correlated IPD^fib^ of fibroblast (for interphase) and IADs of fibroblast, lung endothelial cells, oocyte and Human Umbilical Vascular Endothelial Cells- HUVECs. As the IAD^fib^ of fibroblast at interphase correlated the most with the IPD of fibroblast at interphase, IPD of fibroblast with interphase was further used for testing the cell type specific correlation.

**Figure 3:**
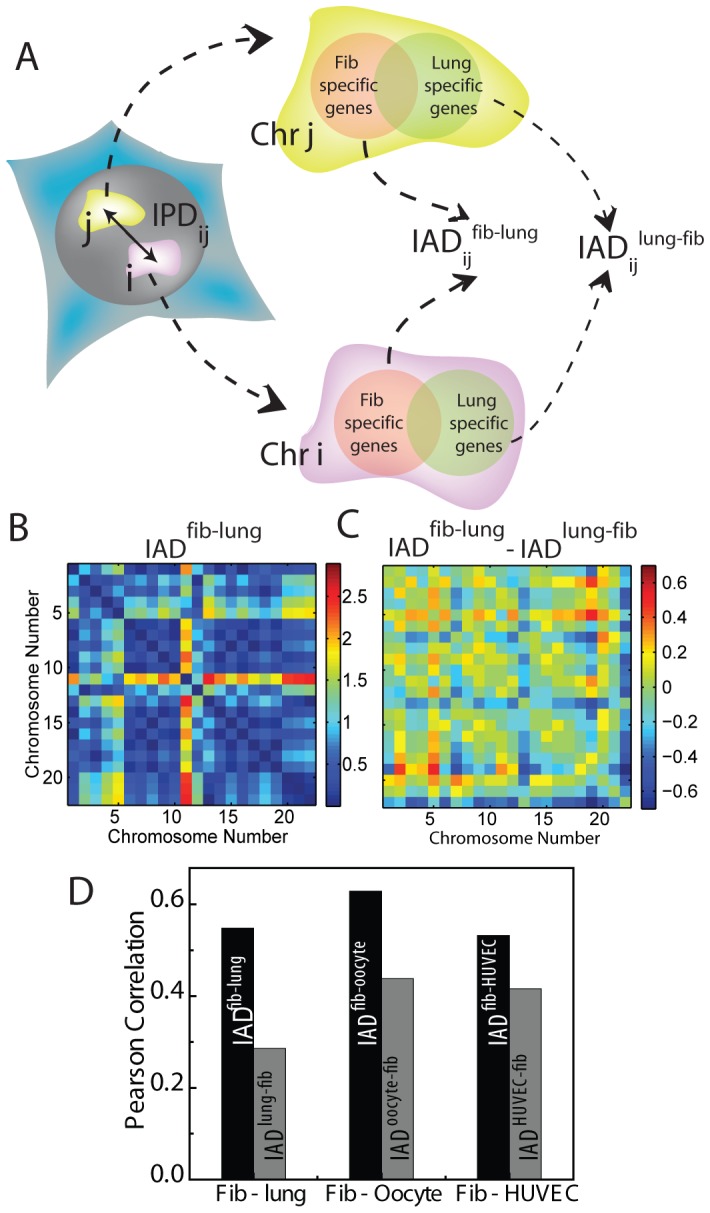
Methods to identify cell-type specific gene expression profiles and its correlation to chromosome positions. (**A**) Schematic description of pairwise comparison of fibroblast with each of the other three human cell types, lung, HUVEC and oocytes. In each comparison, the IPD^fib^ specific for fibroblast is correlated with IAD^fib-other^ of fibroblast, which sums genes expressed in fibroblasts and not in the other cell type, and with IAD^other-fib^ which includes genes expressed in the other cell type (lung, oocyte or HUVEC) and not in fibroblasts. (**B**) IAD^fib-lung^ (in arbitrary units) matrix for genes specifically expressed in fibroblast when compared with lungs. (**C**) Difference matrix (IAD^fib-lung^ – IAD^lung-fib^, in arbitrary units) enhances genes differentially expressing in lungs as compared to fibroblast (**D**). Pearson correlation coefficients (PCC) for the correlation between fibroblast nuclear organization IPD^fib^ and the expression distances IAD^fib-other^ and IAD^other-fib^, for each of the comparisons with the other cell types, lung, HUVEC or oocyte.

From the transcriptome of different cell types, cell type specific genes were selected by excluding similarly expressing genes in pair wise comparison with fibroblasts to generate IADs (Figure 3A). Two activity matrices were generated for each pair of cell type compared: (a) IAD^other-fib^- computed from the activity of genes in the other cell type which are differentially expressed in comparison to fibroblasts (Methods) and (b) IAD^fib-other^- computed from activity of the same genes selected above, in fibroblast. Such activity matrices were computed for each of the three pairs, fibroblast-lung (Figure 3B), fibroblast-oocyte and fibroblast-HUVEC (Methods, Table S2, and Figure S6A & C). Figure 3C depicts the difference matrix of IAD^fib-lung^ and IAD^lung-fib^ for the differentially expressed genes of fibroblast and lung cells. Figure S6B & D shows difference matrices for other cell type pairs. The PCCs were higher when IPD^fib^ was correlated with IAD^fib-other^, whereas the PCCs were comparatively smaller when IPD^fib^ was correlated with IAD^other-fib^ (Figure 3D, Figure S6, Figure S7, Table S1).

These observations suggest that the association between the chromosome topologies and transcription maps is indeed cell type-specific. Similarly, higher PCC were obtained when IAD^fib-other^ were correlated with 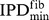 computed from minimum distances between chromosomes and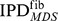 obtained from the multi-dimensional scaling (MDS) of chromosome positions provided by Bolzer *et al.* as against correlations obtained with IPD^other-fib^ (Methods and Figure S8).

### Numerical simulation to probe the coupling between chromosome positions and transcription factor networks

**Figure 4:**
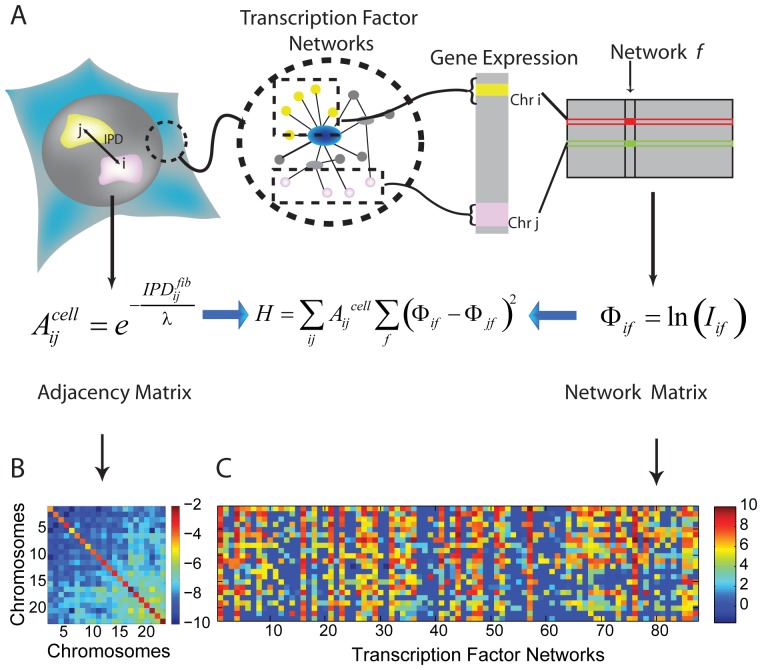
Numerical simulation to probe coupling between chromosome positions and TFNs. (**A**) Schematic description of how the function *H* is calculated by generating the adjacency matrix, *A* ^fib^, from the inter-chromosome distances IPD^fib^ of fibroblast nuclei, and the Network matrices, Φ^cell^ *_if_*, from expression of genes controlled by the various transcription factors, *f*, in cell-type “cell”, sorted into chromosomes *i*. (**B**) Color coded representation of the Adjacency matrix A^fib^ *_ij_* = exp (−IPD^fib^ *_ij_*/λ), between chromosomes in interphase fibroblasts (logarithmic scale). (**C**) Representation of the network matrix Φ^fib^ *_if_* for 87 TFNs in fibroblasts (in arbitrary units).



Genome-wide chromatin interaction experiments have suggested preferential association of genes co-regulated by similar transcription factors [32]. Such *cis-*(same chromosome) and *trans*-(different chromosome) associations have also been shown for co-regulated genes at post transcriptional level at other nuclear bodies [33], [34], suggesting spatial association of genomic elements to facilitate function. In order to probe such associations we devised an energy optimization function and numerical simulation technique which linked the chromosome positions (IPD) to the co-regulated TFNs. In particular, we examined to what extent two chromosomes participate in the same transcription network, tend to be close by. For this purpose, we constructed a function *H*, which measures if nearby chromosomes contain co-regulated genes which belong to a particular functional transcription factor network (Figure 4A). This approach eliminates the whole chromosome averaging that we performed while computing the correlations between IPD and IAD, and considers the activities of only genes which are regulated by a particular transcription factor. However, the position information of genomic elements currently available are at the resolution of chromosomes, leading to coarse graining of this energy optimization function at similar length scales. *H* takes into account both spatial arrangement of chromosomes and the activity of the 87 known annotated TFNs [29], and quantifies how well they correspond to each other. The spatial part of *H* is represented in terms of an adjacency matrix (Figure 4B),

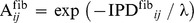



The parameter λ, is the distance parameter used to scale the distances to the length scale of chromosomes. The part of H which involves the contribution from transcription factor networks is introduced as a network matrix (Figure 4C) which is defined as, 
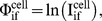
where 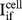 is the integrated microarray intensity of genes present in the *i-*th chromosome that participate in network “f” in cell-type “cell” of the four cell types. Similar to definition of the IAD, logarithmic scale captures the different orders of magnitudes of gene expression. The TF networks which form the network matrix vary from very small networks (<10 genes) to large networks (>300 genes, Figure S9A). To characterize the TF networks for variability in their sizes, we computed the occupancy of chromosomes for each TF network (Figure S9B), which is defined as the fraction of total number chromosomes which have at least one gene from the TF network. Large TFNs have occupancy ∼1 suggesting that the target genes of these TFNs are scattered throughout the genome, whereas, smaller TFNs (<50 genes) have occupancy <0.5, suggesting that their target genes are clustered on a few chromosomes. But this clustering of genes of a TFN are not biased by chromosome size, i.e the genes are present on smaller as well as larger chromosomes (Figure S9A). Further larger chromosomes and gene rich chromosomes were observed to be associated with a larger number of TFNs (Figure S10 and Table S3). The function *H*, which has contributions from spatial organization of chromosomes and the activity of transcription factor networks, is given by,
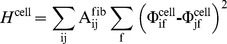



*H* is obtained by summing over all networks *f* for all possible pairs *i-j* of chromosomes, weighted according to the proximity of the chromosomes provided by the adjacency matrix, 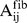. The distance parameter λ, weights the IPD values, such that smaller IPD values attain larger adjacency and vice versa. Moreover λ makes a sharp distinction between nearby and distant chromosomes. For each pair *i-j* of chromosomes, we examine the similarity in the expression levels of genes that belong to a certain network *f*, by summing their squared difference 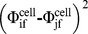 (this ensures the contribution from each pair is positive) and tends to zero for similar activity. The matrices are defined such that when the organization of the chromosomes is correlated with the activity of transcription factor networks, adjacency matrix tends to larger values, whereas 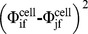
approaches smaller values. In this condition H is defined such that it attains a minimum value. Any deviation from the optimal configuration results in pairing of large adjacency values with large values of 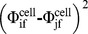 leading to an increase in the value of H.

**Figure 5:**
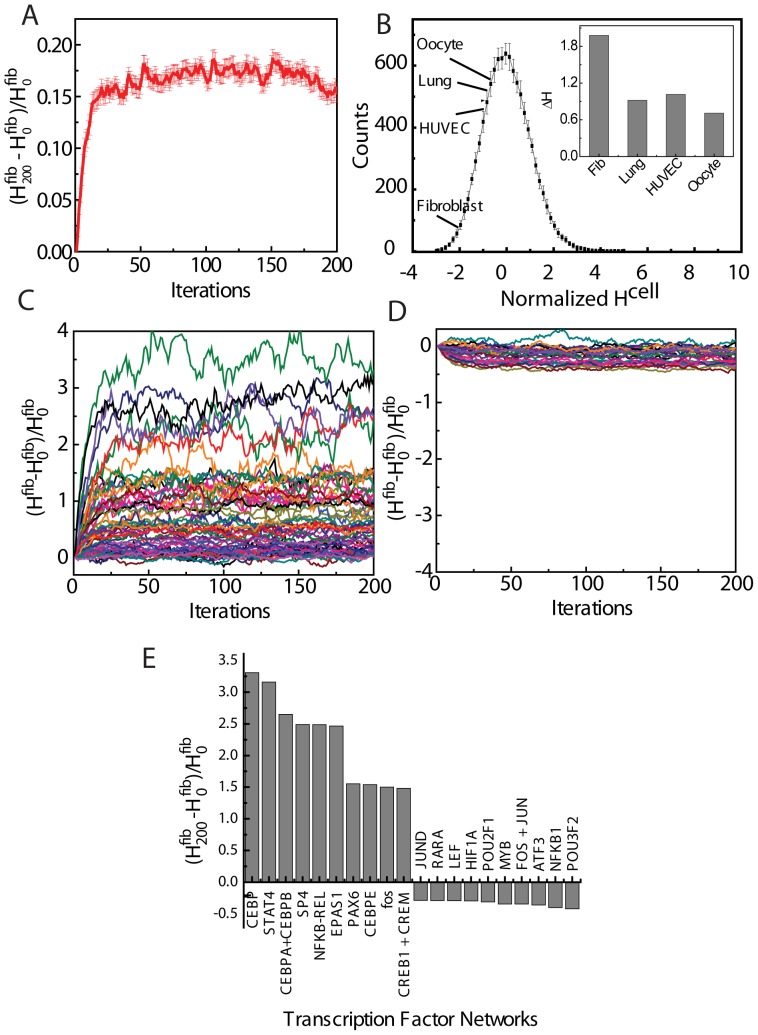
Computation of function *H* to measure the correlation between physical organization (IPD) and network activity (Φ). (**A**) Evolution of H value when the adjacency matrix A^fib^ *_ij_* is randomized. (**B**) The normalized histogram represents the distribution of the *H*-Values for all four cell types. **Inset** shows deviations Δ*H* ^cell^ of *H* ^cell^_0_ from average random *H* ^cell^_av_ measured in units of standard deviation *σ* (**C, D**) Differential changes in the evolution of H value for individual TFNs (c-increase, d-decrease). (**E**) Selected TFNs which show large increase and decrease in H-value. Error bars indicate S.E.M over 20 simulations.

We used numerical simulations to test the above hypothesis. Before performing the actual simulations, we estimated the optimal value of the distance parameter, λ to be ∼7% of the nuclear radius, and it provided the largest increase in the value of H (Figure S11A). We used this distance parameter for all the numerical solutions. To probe the optimality of the value of H, we simulated different configurations of chromosome organization by randomizing the adjacency matrix. The randomization was performed by progressively randomizing the position of pairs of chromosomes by swapping the rows and the columns of the adjacency matrix. H^cell^ was computed after each step of randomization, for 10^4^ iterations. H^cell^ increases upon randomization mostly during the first 200 steps (Figure 5A). Histogram of the 10^4^ recorded *H* ^cell^ values, normalized to its mean 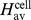 and standard deviation *σ* showed a similar range of H values for all the cell types with H_0_ differing with cell type. (Figure 5B and Figure S11B). A common measure of optimality is the deviation of initial, actual value 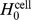 below the average random value,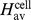, taken in units of *σ*, 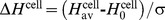. Large deviations indicate that the actual configuration is optimal.

Following this procedure, we find that Δ*H* ^fib^ (ΔH^cell^ for fibroblast) for fibroblast is 1.97, implying a p-value of 0.05 (Figure 5B, inset). This indicates a rather small probability of obtaining a superior configuration through random reorganization of chromosomes in fibroblasts. The network matrix of a given cell type represents its characteristic transcriptional program and the resulting transcriptome. One therefore expects that the coupling between the physical organization and transcription networks will be cell type-specific. Since the spatial part of *H* is taken from fibroblasts, namely the adjacency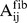, it should exhibit better fit to fibroblast network activity,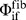, than to the activity of the other cell types. In accord, the deviations for the other three cell types were lower than that of fibroblasts, the values being Δ*H* ^lung^ = 0.92, Δ*H* ^HUVEC^ = 1.02 and Δ*H* ^oocyte^ = 0.71. These correspond to p-values of 0.35, 0.31 and 0.48, respectively, indicating that H values obtained for different cell types are not significantly different from the values obtained for a random configuration of chromosomes. Further, the obtained p- values were independent of the mode of simulation; exclusion of homologues from the simulation and non cumulative randomization resulted in similar p values (Figure S12 and Methods). These results suggest that IPD^fib^ of fibroblast fits better to its own transcriptomes than to those of other cell types.

To analyze the sensitivity of individual TFNs, H values were computed by randomization of the adjacency matrix for the chosen network. The evolution of the H value for the first 200 iterations was plotted for each network. ∼70% of networks showed an increase in the H value (Figure 5C) whereas remaining 30% showed a small decrease (Figure 5D). This indicates the differential contribution of the networks towards optimization of the coupling between TFNs and chromosomal organization. Larger increase in H value for 70% of the networks is in accordance with increase in the value of H when all networks are considered. Figure 5E shows a list of networks that exhibited maximal changes in H values, indicating its sensitivity to perturbations in chromosomal positions. To probe the contribution of TF networks towards differential increase in the value of H, we correlated the change in H value, ΔH/H_0_ with the number of genes in the TF network considered for simulation (Figure S13A). It was observed that ΔH/H_0_ and the number of target genes of a TF network were inversely correlated with decreasing degree of correlation with increase in the distance parameter λ (Figure S13B). As previously observed, the occupancy of the chromosomes has an exponential dependence on the number of target genes of a TF network. This indicates that a large increase in H results from small TFNs, with target genes clustered over a small number of chromosomes. These results indicate that large TFNs which have genes present on all the chromosomes probably regulate housekeeping genes and hence do not contribute strongly towards cell type specific responses.

### Experimental validation

**Figure 6:**
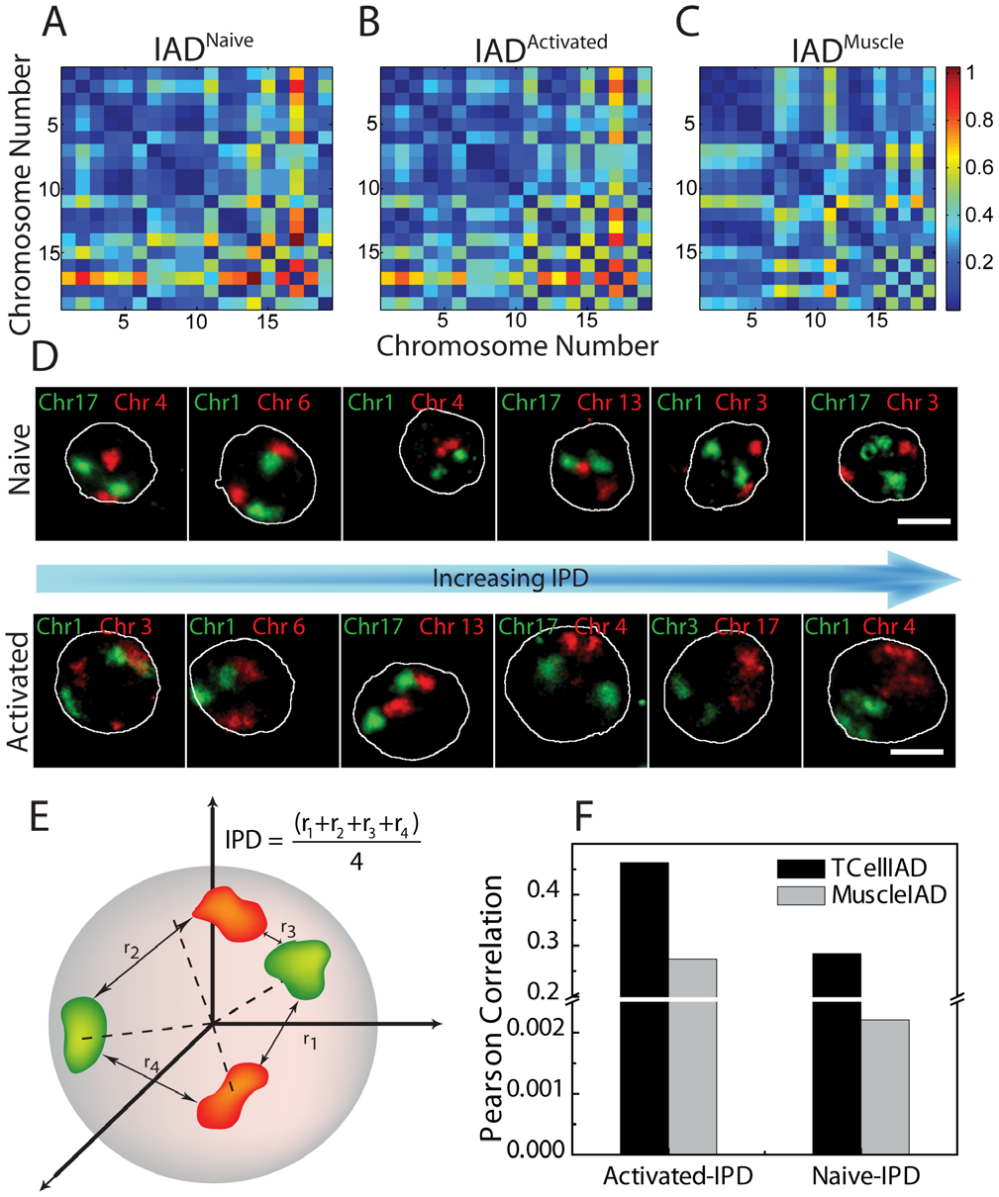
Experimental validation of the coupling between spatial organization and activity in Mouse T cells. (**A**) IAD^Naive^ matrix for murine naïve T cells. (**B**) IAD^Activated^ for activated T cells. (**C**) IAD^Muscle^ for muscle cells. (**D**) Representative images of different pairs of chromosomes (red and green) in naïve (upper panel) and activated T-Cells (lower panel) with DNA in blue, scale bar ∼5 µm. The chromosome pairs are arranged in increasing order of mean IPD values. (**E**) Schematic depiction of estimating the mean IPD from the four minimum distances between the interfaces of the four homologues of a pair of chromosomes. (**F**) Pearson correlation coefficients (PCC) computed for IAD of T cells or muscle cells with IPD of T cells.

Our numerical approaches suggested that spatial arrangements of chromosomes in a given cell type (human fibroblasts) is optimized to its expression pattern better than it fits to the expression patterns in other cell types. To further validate the results from proposed numerical approaches, we experimentally tested the correlation between chromosome positioning and gene expression in another cell type of a different mammal, murine T cells, in two distinct transcription states of naïve and *in vitro* activated T cells, where the global mRNA levels increase by ∼5 fold [35]. We generated IAD for both the naïve and activated T cells (IAD^Naive^ and IAD^Activated^) from genome-wide microarray data (GEO accession number GSE30196) obtained from our experiments (Figure 6A & B). The microarray was done in duplicates. IPD was estimated for candidate pairs of chromosomes 1–3, 1–4, 1–6, 3–17, 4–17 and 13–17 (which harbor 30% of the differentially expressed genes identified in the microarray) by 3D FISH performed in naïve and activated T cells (IPD^Naive^ and IPD^Activated^) (Figure 6D). The cells used for estimation of IPD were obtained from different batches of cell purification, using similar methods as for IAD estimation. The homogeneity between the cells isolated from two different mice was quantified by comparing differences in number of differentially regulated genes at similar conditions from two biological replicates. Figure S14 shows that the number of differentially regulated genes are more than ten folds higher in between different states of T Cell (e.g. naïve NC1 and activated NC2) isolated in same batch when compared to the number of differentially regulated genes, between biological replicates of same cell type (e.g. naïve NC1 and naïve NC2). IAD matrices computed from the biological replicates also showed very small variations (measured as matrix of standard deviation between IAD values generated from two replicates) indicating that different batches of cells does not significantly alter the IAD matrix (Fig S15). The IPD which we used in earlier correlations, was generated from the centroid positions of the chromosomes obtained from 3D chromosome FISH (Fig 6D and Fig S16). Inter-centroid distances are biased by the size of the chromosomes. Two large chromosomes will tend to have their centroids farther apart as compared to small chromosomes, even though the distance between the chromosome surfaces may be same. To overcome this drawback, we generated the IPDs for specific pairs of T Cells by measuring the distance between the chromosome surfaces. The IPDs were generated using minimum interface distance among all the four possible interface distances between the pair. (Figure 6E) The IPD of Naïve and Activated T cells were correlated with their respective IADs and IAD^Muscle^ of murine muscle cells (Figure 6C). The obtained PCC values using IPD and IAD (Table S4) of both naïve (0.28) and activated states (0.46) were higher in contrast to PCC computed between IAD^Muscle^ and IPD of T cells (0.002- IPD^Naïve^, 0.27-IPD^Activated^). Interestingly, the differences in the correlation coefficients are similar (0.28−0.002 = 0.278 and 0.46−0.27 = 0.19) for both naïve and activated T cells when compared to muscle cells. These results indicate that correlation between chromosome organization and their transcriptional output is a general phenomenon which can be observed in multiple cell types.

## Discussion

Random loop polymer models have been extensively used to understand the internal architecture of chromosomes. These studies suggest a gene expression based looping probability of the chromatin fibre which leads to formation of functional DNA domains and confinement of chromatin to chromosome [36]–[38]. Transcriptional activity based chromosome intermingling has also been used to explain the frequent juxtaposition of certain pairs of chromosomes and the resulting chromosomal translocation [14]. But very few methods are present to quantitatively measure the correlation between the physical proximity of chromosomes and transcriptional activity [16]. In this work we propose methods to probe the correspondence between the chromosome positions and global gene expression program. While chromosomes have been found to be radially distributed from the nuclear centroid according to their gene density [39]–[41], our methods were able to assess a further layer of three dimensional organization, in which the relative chromosome positions correlated with gene expression. Previous studies suggest both random[42], [43] and non random [27], [44], [45] chromosome positions, whereas our results and analysis revealed the non-random organization within the fibroblast nucleus [27]. The IADs computed from the microarray data showed correlation between relative chromosome activities and their respective positions. These correlations support the co-clustering of genes for transcriptional control [21] by a fewer number of observed transcription factories [13], [15]. The observed correlations may also have contribution from noise due to coarse graining and population averaging of the chromosome positions and their activities. Further, noise in chromosome activity measurements could also be contributed by the matured mRNA which does not exactly represent the stochastic nature of short lived nascent mRNA transcripts produced at the sites of transcription at single cell level [46], [47]. The correlations can be improved by extending the methodology described here to build a more detailed IPD for smaller continuous regions of the chromosome and their corresponding IADs. Our evaluation methods in different cell types suggested that arrangement of co-clustered [48] genes must be cell type specific as we find a lack of correlation between chromosome positions of one cell type with the gene expression program of another cell type . The cell type specific transcriptional programs are usually turned on by cell type specific TF networks [49], suggesting their involvement in modulating inter-chromosome interactions. Previous simulation and modeling work on the role of transcription factors in organization of genome in *E. coli* suggested formation of DNA regulatory domains of co-regulated TF target genes [50]. Similarly in yeast, target genes of TFs were shown to be preferentially co-clustered on the same chromosome [51]. In this work we have taken this idea further to suggest role of TF networks in elucidating relative chromosome proximities in nucleus. Numerical simulations shown here suggest that the activity of TFNs was correlated with relative positions of chromosomes. An optimality measure H was devised to quantitatively understand the coupling between 3D chromosome positions and TFNs. Our predictions of the correspondence between chromosome positions and global gene expression were experimentally validated in naïve and activated states of mouse T-cells. These results evidenced correlations between the IPD and IAD of T cells whereas smaller correlations were observed between IPD of T cells and IAD of muscle cells.

Taken together, our methodologies were able to quantify correspondence between global gene expression program and three-dimensional architecture of chromosome positions. While co-clustered genes have been shown to be co-regulated [21], [52], [53], methods proposed here take these findings to the large-scale organization of the nucleus where transcription dependent intermingling of proximal chromosome territories may become feasible. Interestingly, these correlations are found both at the scale of transcriptome and at the scale of separate transcriptional networks [54]. Our findings suggest that the observed correlations between relative chromosome positions and transcriptional output are specific to a given cell type. The measured correlations are at steady state and with time averaged expression profiles, which smears the time resolved correspondence between chromosome positions and transcriptional activity. However, mechanistic insights into the origin of such correlations could be gained if such correlations are observed during the process of differentiation. Such refinements in IPD and IAD at single cell resolutions can in future yield better insight about contribution of transcription in relative chromosome organization. In addition, the chromosome position in the nucleus is a result of integration of many functional and spatial organizational cues like epigenetic modifications [55], transcription machinery density, post transcriptional or replication requirements. The methodology presented here can be easily adapted to further investigate the contribution of these factors by quantifying them at similar resolution as IPD for chromosomes. The general mechanisms of chromosome topology [11] and their functional links will become apparent as one simultaneously probes the temporal evolution of these correlations through the process of cellular differentiation and its maintenance through cell cycle.

Current methods to evaluate chromosome positions and its impact on gene expression have remained empirical. The introduction of 2D matrix for chromosome positions enabled an analysis of transcriptional changes through cellular differentiation [16]. Our methods further establish the coupling between chromosome positions optimized for a given cell type on a quantitative framework. By implementing comparative analysis methods between chromosome position matrix and activity matrix we were able to evaluate the coupling between TFNs and chromosome organization. Our proposal of a phenomenological analytical function (H), allow a systematic numerical simulation of correlations relating the TFNs and chromosome topology. The function H could be modified to include epigenetic modifications or active RNA polymerase interactions to construct an activity distance matrix of these parameters. This method could be adapted to chromosome sub-domains by painting smaller regions of chromosomes or using contact probabilities from chromosome capture assay [11] and correlating them with the corresponding activity distance matrix of these parameters. These matrices may further provide correlations at a finer resolution of gene clusters and their correspondence with transcription. We suggest that our methods describing interfaces of CTs in conjunction with chromosome capture assays may also facilitate identifying cell type specific functional gene clusters. The methods described in this work could also be useful in establishing correlations between three dimensional organizations of chromosome positions with other functional networks like signaling networks and chromatin remodeling networks.

## Methods

### Ethics Statement

All experiments involving animals were performed with the approval of the Institutional Animal Ethics Committee at National Centre for Biological Sciences, Bangalore headed by Prof. Mathew with committee members Professors Upinder Bhalla, Sumantra Chatterjee, MM Panicker and R. Sowdhamini. Approval ID for the project is AS-5/1/2008.

### Inter-chromosome distance (IPD)

The Inter-chromosomal Physical Distance (IPD^fib^) for fibroblast was obtained from the chromosomal distances as:
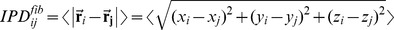



Where *r_i_* and *r_j_* are the chromosomal distances from the nuclear centroid (in units of nuclear radius) in case of interphase and from centre of the prometaphase ring in case of the prometaphase chromosomes, obtained from Bolzer *et al.* [27]. Each element in the matrix is calculated from the mean of four possible distances between two pairs of homologous chromosomes (as the current experiments cannot distinguish between two different homologues of same chromosome), and further averaged over the 54 nuclei. Similar IPD matrices were constructed using the MDS distances (IPD_MDS_) and minimum distances between the four possible values between two pairs of homologous chromosomes (IPD_Min_).

### IPD Randomization procedure

The interchromosomal physical distance, IPD^rand^ for a random configuration of the nucleus was obtained from iterative swapping of the chromosomes in the fibroblast nucleus, by shuffling the rows and columns of the IPD^fib^ matrix for 200 iterations (Figure S1), which was sufficient for complete randomization, i.e. loss of chromosome position information from the initial configuration. The randomization process was designed to obey the triangle inequality, a basic property of Cartesian metric space, as no new spatial coordinates are created other than the actual r_i_ already present. Rather, the rows and columns of the IPD matrix were interchanged in a cumulative fashion, with each shuffling performed on the previously shuffled matrix.

### Inter-chromosome Basepair length Differences (IBD) Matrix

Differences in chromosome basepair length size was represented as Inter-chromosome Basepair length difference (IBD) matrix defined as



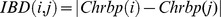



Where *Chrbp(j)* and *Chrbp(j)* are the basepair lengths of chromosome i and j respectively.

### Inter-chromosome Activity (IAD) Matrices

The Inter-chromosome Activity Distance (IAD) was created from the microarray data of fibroblast, obtained from Goetze *et al.* [28] (GEO accession no.-GSM157869). In the microarray there are multiple probes for a single gene. The activity of a gene in arbitrary units was obtained by calculating the mean intensity of the multiple probes for a given gene. The genes were further grouped into individual chromosomes and the activities were integrated over the whole chromosome. The total number of genes in each chromosome was obtained (http://vega.sanger.ac.uk/Homo_sapiens/index.html, as on Nov 11, 2008) and used to estimate the mean chromosome activity as:
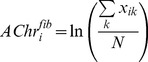



Where *x_ik_* denotes the activity of k^th^ gene in the microarray for chromosome *i* and N is the total number of annotated genes in a chromosome with summation done over all genes in the microarray for a particular chromosome. Logarithmic activities were obtained for each chromosome to account for the large dynamic range of the gene expression data. The IAD matrix was generated from the logarithmic chromosome activity as:
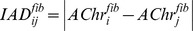



To generate the IAD matrices for additional cell types the microarray data for lung, oocytes and HUVEC were obtained from Gene Expression Omnibus website (http://www.ncbi.nlm.nih.gov/geo). The accession number for the datasets are GSM101102 (lungs cells) [56], GSM288812 (oocytes) [57] and GSM215557 (HUVECs) [58]. The chosen microarray data was performed on Affymetrix GeneChip and MAS5 algorithm was used to calculate probe intensity, identical to that of fibroblast microarray data. In order to normalize the intensity variation between different cell types the microarray data was normalized to the mean of the probes in the array.



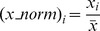
where *(x_norm)_i_* is the normalized activity of the probe and 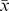 is the average activity of the probes in the array. The activity of a gene was calculated by taking the mean of the normalized probe intensities. To evaluate cell-type specific activities, cell types were considered as pairs (fibroblast-lung, fibroblast-HUVEC and fibroblast-oocyte). For example, if fibroblast and lung is considered as a pair, the differentially expressed genes between the two cell types were selected based on the expression level differences of corresponding genes in the two cell types. Genes with expression level difference of more than 1FWHM (Full Width at Half Maxima, calculated from the difference histogram) were selected. Hence, the activities of same genes from both cell types were compared. Further, these genes were partitioned into their respective chromosomes and the IAD matrix for each cell type was computed with their respective logarithmic activities (similar to IAD^fib^). The two resulting matrices were named as IAD^fib-lung^, if activity is computed from fibroblast microarray data and IAD^lung-fib^, if the activity is computed from microarray of lung cells. This ensures that the number of genes selected for a given chromosome is same in both the cell types

### Adjacency and network matrix

The physical space of chromosomes was represented by the IPD^fib^ matrix. To enhance the sensitivity of the chromosome positions, an adjacency matrix was generated with weights for each inter-chromosome distance as:
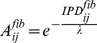



Where, the λ in the exponential is the distance parameter which was varied from 2–80% of typical nuclear radius, to find the optimum λ. Upon variation of λ, the increase in H-value was computed (Figure S7) and the λ which showed maximum increase was selected as the optimal λ for the simulation. This functional form of adjacency matrix with a steep slope is sensitive to change in chromosome positions. The exponential form was used to detect small deviations in chromosome organization from the optimum configuration. The network space was represented by a network matrix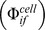, consisting of 87 annotated transcription factor (TF) networks obtained from Transcription Regulatory Element Database database (TRED), Jiang *et al* [56]. The genes in the TF network were identified in the microarray data for human fibroblast, lung, oocyte and HUVEC cell types and grouped into chromosomes. Chromosomal activity of genes involved in a particular TF network was obtained by calculating the natural logarithm of the integrated activities of all the identified genes in a chromosome. The chromosomes in which no genes were identified, a value of zero was assigned for that chromosome in the network matrix. A column vector was created for individual networks,
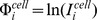
where column *f* represents single TF network. The 87 column vectors were aligned to obtain the network matrix for the TF network,
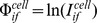
where *f* is a network and *i* a chromosome.

All abbreviations used are listed in Table S5.

### Mouse T-cell Microarray and Analysis

For T-cell experiments, cells were isolated from spleens of C57/Bl6 mice. All animals were bred and maintained in the NCBS animal house facility. Experiments were performed with the approval of the Institutional Animal Ethics Committee at NCBS, Bangalore, India. CD4^+^ naïve T-cells were isolated from spleen of 8–10 week old mice using MagCellect isolation kit (R&D Systems, MN, USA) activated for 36 hours using αCD3-αCD28 coated beads (Invitrogen, CA, USA). This method consistently produces 90–98% pure populations of T cells (according to manufacturer's protocol). Microarray experiments on cells, purified in our laboratory at NCBS, Bangalore, were carried out at Genotypic (Bangalore, India). Duplicate experiments were done for both naïve and activated T cells. RNA extraction was done using RNeasy Minikit (Qiagen, UK), concentration and purity were determined using Nanodrop® ND-1000 spectrophotometer (NanoDrop Technologies, Wilmington, DE, USA). The integrity of RNA was verified on an Agilent 2100 Bioanalyzer using the RNA 6000 Nano LabChip (Agilent Technologies, CA, USA). Equal amounts of RNA was labeled using Agilent dye Cy3 CTP and hybridized to *Mus musculus* GeneExpression Array 4X44K (AMADID -014868). The slides were scanned using Agilent Microarray Scanner G2505 version C at 2 µm resolution, and data was extracted using Feature Extraction software v 9.5 of Agilent. Though the microarray was performed on two batches of T cells obtained from two different mice, the gene expression across replicates was very similar as compared to the gene expression between naïve and activated T Cells, and hence it does not introduce significant noise in the analysis.To further minimize the noise in estimation of genome wide expression profile, mean of probe intensity for two duplicates was computed. Mean of intensities of probes for the same gene was calculated to obtain the activity per gene. Further, genes were grouped into chromosomes and IAD matrix was computed as explained earlier. The microarray data is MIAME compliant and accessible on GEO website (http://www.ncbi.nlm.nih.gov/geo/; accession number GSE30196).Microarray data of murine muscle cells was obtained from Gene Expression Omnibus (GEO accession number GSM247205) and used as a negative control.

### Chromosome painting and image analysis

For chromosome painting experiments, cells were stuck on PDL coated slides followed by fixation in 4% PFA for 10 minutes. PFA was neutralized with 0.1M Tris-HCl and then the cells were washed and permeabilized with 0.5% Triton X-100 for 8 minutes. This was followed by incubation in 20% glycerol for 1 hour and then 5 or 6 freeze-thaw cycles in liquid nitrogen. After this, cells were treated with 0.1N HCl for 10 minutes, washed and equilibrated in 50% Formamide/2X SSC overnight at 4°C. Hybridization was set up the following day. Cells were denatured in 70% formamide/2X SSC at 85°C for 2 minutes and then incubated with the fluorescently labeled mouse whole chromosome FISH probes (Cambio, Cambridge, UK) for 2–3 days in a moist chamber at 37°C with shaking. At the end of the incubation period, slides were washed thrice each in 50% Formamide/2X SSC at 45°C and 0.1X SSC at 60°C. Cells were counterstained with Hoechst 33342 (Sigma, USA) and then mounted with Vectashield (Vector Laboratories, CA, USA), sealed with coverslip and imaged on a Zeiss 510-Meta confocal microscope.

Inter-chromosome interface distances were computed using a custom written program in LabVIEW (National Instruments, TX, USA). Confocal Z sections for each chromosome were thresholded using (mean+standard deviation) of the fluorescence intensity of the Z-stack. Edge detection algorithm was used on each thresholded confocal section to obtain coordinates of chromosomal edge at each z plane. The three dimensional distance between the edges of two chromosomes were computed. The interface distance between two chromosomes was estimated by calculating the minimum distance, out of all the distances computed between the edge coordinates of the two chromosomes. Since two pairs of chromosomes are labeled in each nucleus, four interface distances were obtained. Average of the four interface distances for a given pair of chromosomes was then used as IPD_mean_ for the given pair of chromosomes.

### Statistical analysis

False discovery rate (FDR) was employed as a statistical measure to test the significance of the correlations obtained (Figures S3 & S5 and Table S2). The actual pearson correlation (PCC) value in each case was denoted as PCC_0_. To compute the FDR, 10^5^ randomized matrices (from either IPD^fib^ or IPD^prometaphase^) were generated (each randomized matrix was computed by permuting its rows and columns) and each randomized matrix was correlated with matrix under consideration. A histogram of all the PCC value for the correlation between the matrices was generated and the instances with PCC>PCC_0_. FDR was estimated as the fraction of instances with PCC>PCC_0_



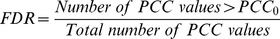



## Supporting Information

Figure S1 **Correlation of IPD and IAD with chromosome size differences.** (A) Correlation between Gene number and Chromosome size in base pairs. (B) Bar graph representing the gene number and chromosome size plotted for gene poor “chromosome 18” and gene rich “chromosome 19”. (C) Interchromosome Basepair length Difference (IBD) matrix for human fibroblasts. (D) Correlation between IPD^fib^ and IBD of fibroblast. (E) Correlation between IBD and IAD of fibroblast. (F) Pearson Correlation Coefficients for IPD-IBD and IBD-IAD correlation.
(TIF)

Figure S2 **Scheme of randomization.** (A) Sequence of color coded matrices representing the difference between IPD_0_^fib^ - before randomization and IPD^fib^ - after every step of randomization. The colors represent the difference between randomized IPD and first IPD_0_^fib^ matrix in units of nuclear radius. Before randomization the values were all zero indicating unrandomized matrix, whereas after several randomizations the matrix becomes randomized. (B) Pearson correlation between IPD_0_^fib^ before randomization and IPD after stepwise randomization (C) PCC between IAD and IPD during stepwise randomization. Error bars indicate S.E.M.
(TIF)

Figure S3 **Correlation between IPD at different cell cycle stages and IAD.** Representative scatter plot and corresponding fit between (A) IPD^prometaphase^ and IPD^rand^ and (B) IPD^prometaphase^ and IAD^fib^ for Human fibroblast. PCC histogram for (C) IPD^fib^ and IPD^rand^ correlation and (D) IAD^fib^ and IPD^rand^ correlation s for 10^4^ different randomized IPD^rand^ matrices
(TIF)

Figure S4 **False Discovery rate estimation.** (A) Histogram of PCC values for correlation between IPD^fib^ and IPD^prometaphase^ for 10^5^ different randomized IPD^rand^ matrices. Gray bars indicate PCC values less than the PCC value of unrandomized matrix (PCC_0_) and red indicates the values greater than PCC_0_. False discovery rate (FDR) is computed as the fraction of PCC values above PCC_0_. Histogram similar to (A) are shown for correlation between IPD^fib^ and IAD^fib^ (B) and for correlation between IPD^prometaphase^ and IAD^fib^ (C).
(TIF)

Figure S5 **Correlation between IPD of fibroblast and IAD of fibroblast from selected genes.** (A) IAD^select^ of fibroblast generated from stringently selected genes with expression higher than 0.4 times the mean expression of the genes in a chromosome. (B) Correlation between IPD^fib^ and IAD^select^. (C) Distribution of PCC for correlation between 30,000 different randomized IPD matrices and IAD^select^. The grey bars indicate PCC<PCC_0_ and red bars represent false discovery with PCC>PCC_0_
(TIF)

Figure S6 **Matrices and scatter plot for comparison of fibroblast with other cell types.** (A) IAD^fib-oocyte^ matrix is generated from the activity in fibroblast for differentially expressing gene between fibroblast and oocyte. (B) Difference between IAD^fib-oocyte^ and IAD^oocyte-fib^, where IAD^oocyte-fib^ is the matrix generated from activity in oocyte for differentially expressing gene between fibroblast and oocyte. (C) and (D) are computed similarly as matrices in (A) and (B) respectively for IAD^fib-HUVEC^. (E-G) Scatter plot and corresponding fits between IPD^fib^ and IAD^fib-other^ or IAD^other-fib^ where in (E) other = lung, (F) other = oocyte and (G) other = HUVEC.
(TIF)

Figure S7 **Estimation of false discovery rates for IPD and IAD correlations for different cell types.** (A–F) Grey lines indicate PCC values less than the PCC value of the unrandomized matrix (PCC_0_), whereas red lines indicate PCC values greater than PCC_0_. FDR is computed as a fraction of the PCC values above PCC_0_. PCC for correlation between IPD^fib^ and IAD^fib-other^ has a significantly smaller false discovery rates (FDR) as compared to the PCC value for correlation between IPD^fib^ and IAD^other-fib^.
(TIF)

Figure S8 **Pearson correlation and slope** **for the correlation between IPD^fib^ and IAD^fib-other^ or IAD^other-fib^** generated from (A) minimum distances (IPD_min_) and (B) MDS distances (IPD_MDS_) provided in Bolzer *et al.*
(TIF)

Figure S9 **Characterization of TF networks.** (A) Distribution of genes of a TF network (with<50 genes) over different chromosomes. The distribution of genes are not biased by size of the chromosome. (B) Occupancy of chromosomes for different TF networks. Occupancy is defined as the fraction of chromosomes having at least one gene from a TF network.
(TIF)

Figure S10 **Dependance of Chromosomal association of TFs on chromosome length and number of genes.** (A) Correlation between numbers of TFNs associated with a chromosome and the length of chromosome in base pairs, with a PCC of 0.53. (B) Correlation between number of TFNs associated with a chromosome and the number of annotated genes on that chromosome (PCC = 0.58).
(TIF)

Figure S11 **Dependence of H-values on the distance parameter.** The variation of (H−H^0^)/σ with variation in the value of λ (in units of % nuclear radius), shows a maximum value at λ = 7% of nuclear radius for all the different cell types. (B) Histogram of H values for 10,000 iterations of randomization, computed for four different cell types.
(TIF)

Figure S12 **Different modes of simulation yield similar p values for different cell types.** (A) p values obtained for estimation of H values without considering the adjacency matrix values for homologues. (B) p values obtained for estimation of H values without cumulative randomization of the adjacency matrix. H values in this case are computed after 100 steps of randomization of the adjacency matrix, for 10,000 iterations.
(TIF)

Figure S13 **Dependance of Change in H value upon the number of genes in a TF network.** (A) Correlation between ΔH/H_0_ and number of genes in a TF network. (B) Pearson correlation coefficient of the correlation between ΔH/H_0_ and number of TF network depends on the distance parameter (λ).
(TIF)

Figure S14 **Similarity in gene expression across batches** **of T- cells**. (A) scatter plot between log2 ratios of NC1 (Naïve T Cell, replicate 1) & NC2 (Naïve T cell, replicate 2) , and log2 intensity of NC1 and NC2, showing that there are very few differentially expressing genes across two different batches of Naïve T cells. (B) Scatter plot similar to (A) between AC1(Activated T cell, replicate 1) and AC2 (Activated T cell, replicate 2). (C) Scatter plot between log2 ratios of NC1 and AC1, and log2 intensities of NC1 and AC1, showing large number of diiferentially expressing genes, when Naïve and Activated T cells of the same batch are considered. (D) Scatter plot similar to (C) between NC2 and AC2. (E) Number of differentially expressing genes when either naïve or activated T cells of different batches are considered (NC1 vs NC2 and AC1 vs AC2), or when naïve and activated T cells of the same batch are compared (Nc1 vs AC1 and NC2 vs AC2).
(TIF)

Figure S15 **Variability in IAD matrix across batches of cells. (**A) Mean IAD^Naive^ matrix averaged over two replicates of microarray from two batches of cells. (B) Matrix showing the standard deviation in the value of IAD^Naive^ matrix. (C) Mean IAD^Activated^ matrix. (D) Standard deviation in estimation of IAD^Activated^.
(TIF)

Figure S16 **3D Chromosome FISH in Naïve and Activated T-Cells.** (A) Images of nuclei showing 6 different chromosome pairs labeled in mouse naïve and activated T-cells. White outline indicates the boundary of the nucleus. (B) Interchromosomal Interface distances in Naïve and activated T cells. (B) Coefficient of variation (σ/µ) computed for the interchromosome interface distances for naïve and activated T cells. Scale bar, 5 µm.
(TIF)

Table S1 **Pearson Correlation and FDR Table.** The table shows the Pearson correlation and the corresponding false discovery rates when **Matrix 1** is correlated with **Matrix 2**
(TIF)

Table S2 **Table of Differentially expressed genes within cell types.** The table represents the total number of annotated genes and the number of genes selected for differential expression between fibroblast and other cell types.
(TIF)

Table S3 **Transcription Factor Network Association with chromosomes.** This table provides the number of Transcription Factor networks associated with different chromosomes.
(TIF)

Table S4 **T-Cell IPD and IAD table.** The table shows the IPD for naïve and activated T-Cell for selected pairs of chromosomes and IAD for Naïve, Activated and Muscle cells for the same pair of chromosomes.
(TIF)

Table S5 **Table of Abbreviations**. This table provides the meaning of the abbreviations and notations used in the manuscript.
(TIF)
