# Supplementary figures and images for: Modeling and Experimental Methods to Probe the Link between Global Transcription and Spatial Organization of Chromosomes
Source: PLoS One. 2012 Oct 1;7(10):e46628. doi: 10.1371/journal.pone.0046628 (PMC3462193; doi:10.1371/journal.pone.0046628)

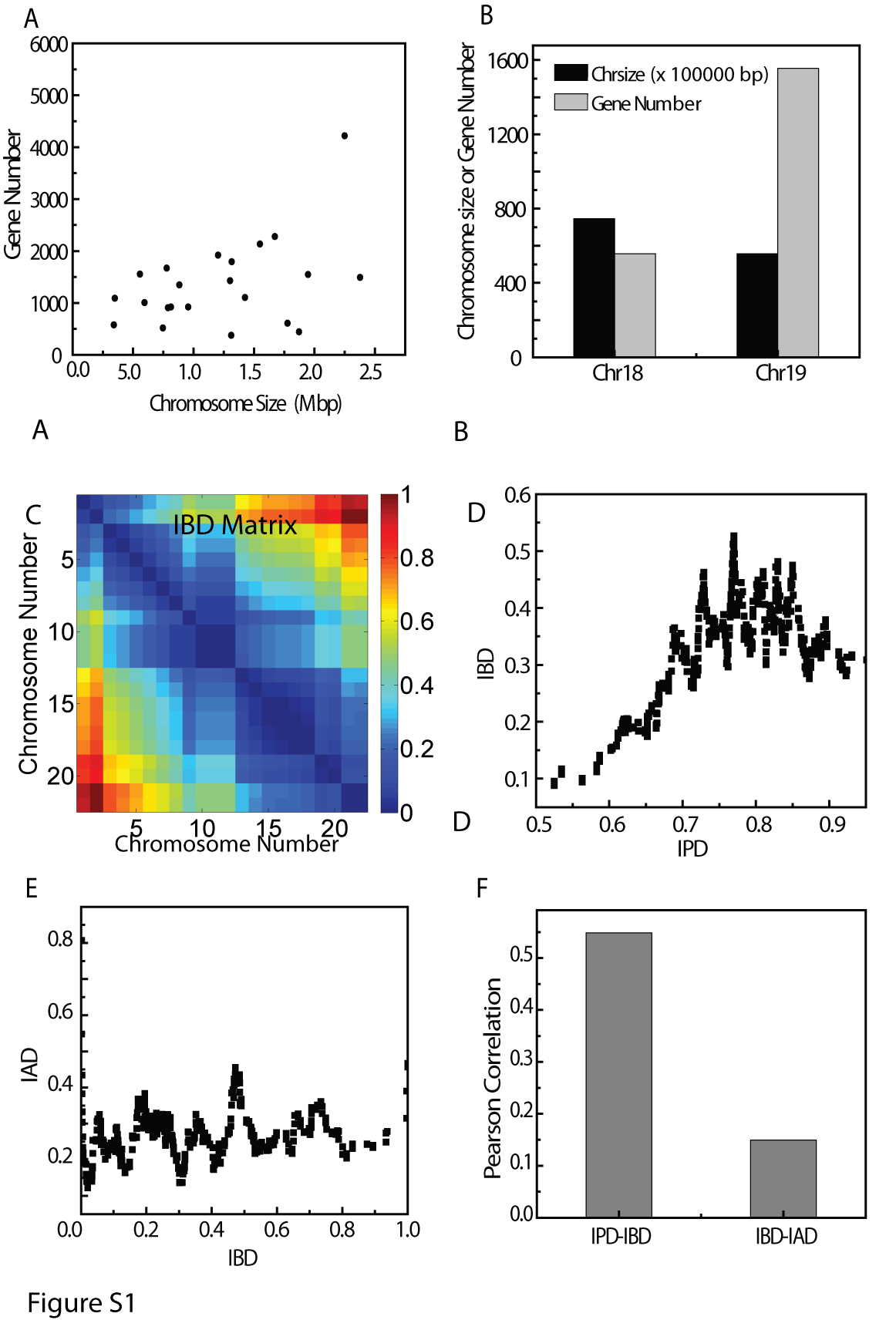

Supplement: Figure S1 — Correlation of IPD and IAD with chromosome size differences. (A) Correlation between Gene number and Chromosome size in base pairs. (B) Bar graph representing the gene number and chromosome size plotted for gene poor “chromosome 18” and gene rich “chromosome 19”. (C) Interchromosome Basepair length Difference (IBD) matrix for human fibroblasts. (D) Correlation between IPDfib and IBD of fibroblast. (E) Correlation between IBD and IAD of fibroblast. (F) Pearson Correlation Coefficients for IPD-IBD and IBD-IAD correlation. (TIF) [file pone.0046628.s001.tif]

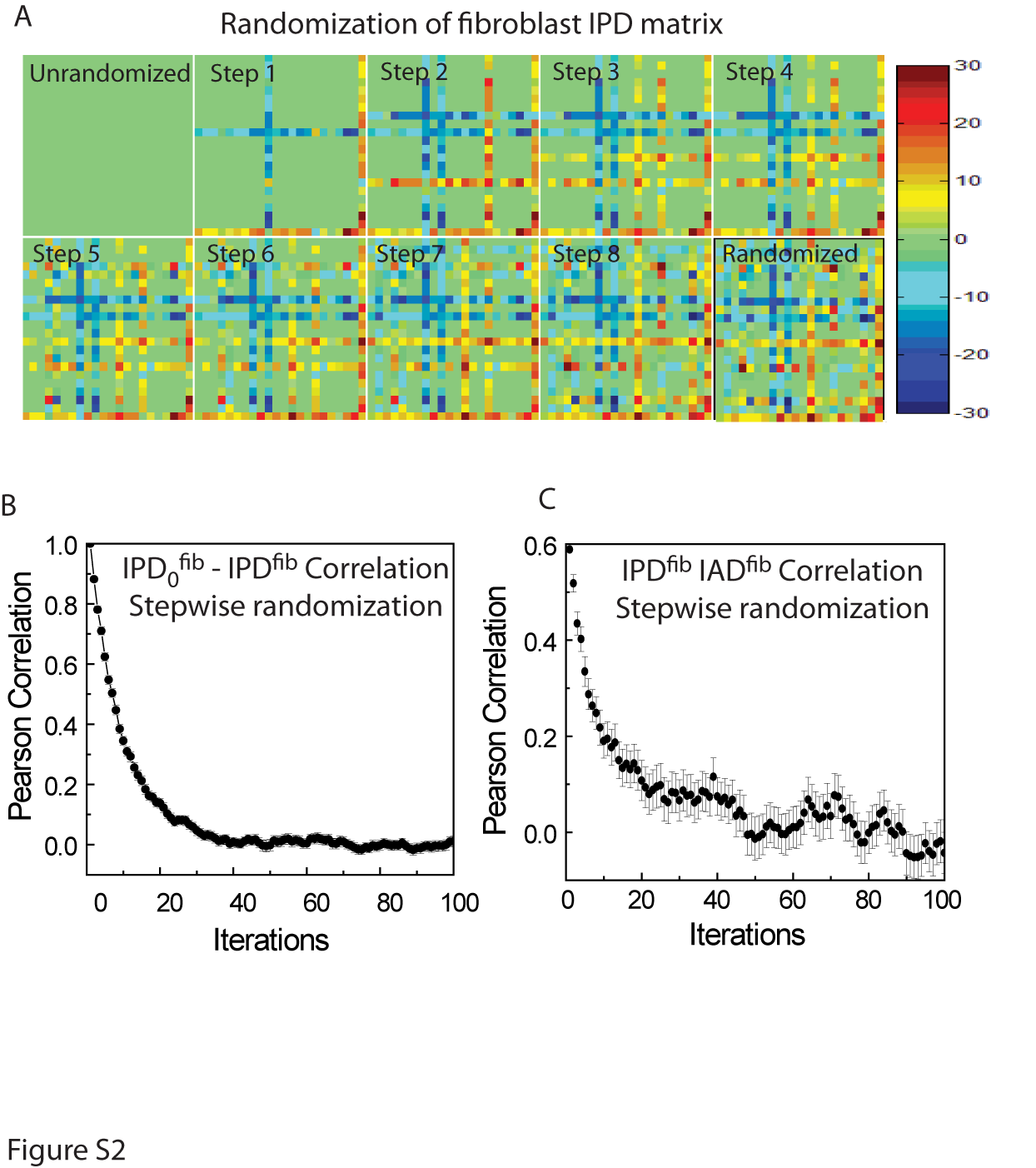

Supplement: Figure S2 — Scheme of randomization. (A) Sequence of color coded matrices representing the difference between IPD0 fib - before randomization and IPDfib - after every step of randomization. The colors represent the difference between randomized IPD and first IPD0 fib matrix in units of nuclear radius. Before randomization the values were all zero indicating unrandomized matrix, whereas after several randomizations the matrix becomes randomized. (B) Pearson correlation between IPD0 fib before randomization and IPD after stepwise randomization (C) PCC between IAD and IPD during stepwise randomization. Error bars indicate S.E.M. (TIF) [file pone.0046628.s002.tif]

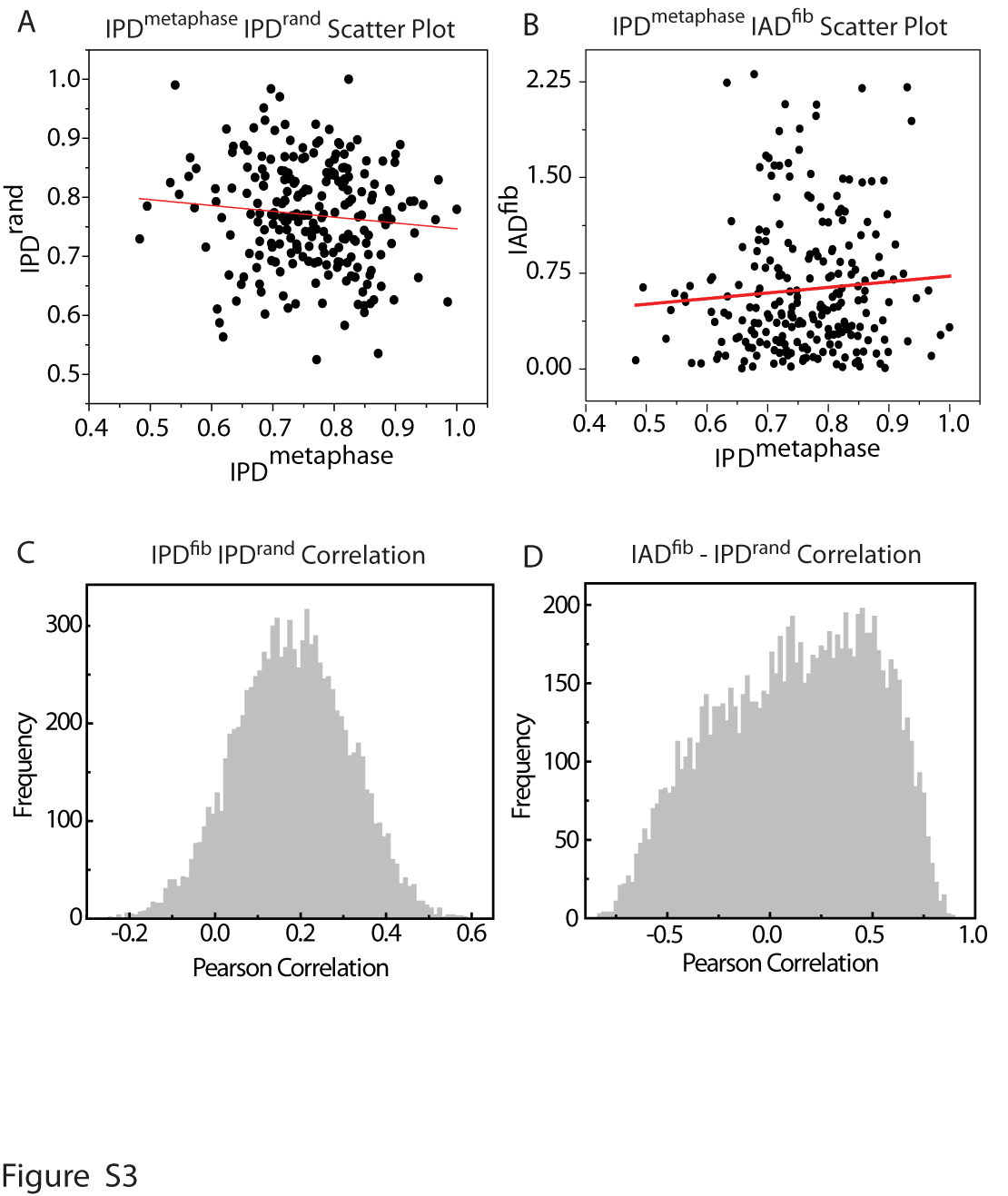

Supplement: Figure S3 — Correlation between IPD at different cell cycle stages and IAD. Representative scatter plot and corresponding fit between (A) IPDprometaphase and IPDrand and (B) IPDprometaphase and IADfib for Human fibroblast. PCC histogram for (C) IPDfib and IPDrand correlation and (D) IADfib and IPDrand correlation s for 104 different randomized IPDrand matrices (TIF) [file pone.0046628.s003.tif]

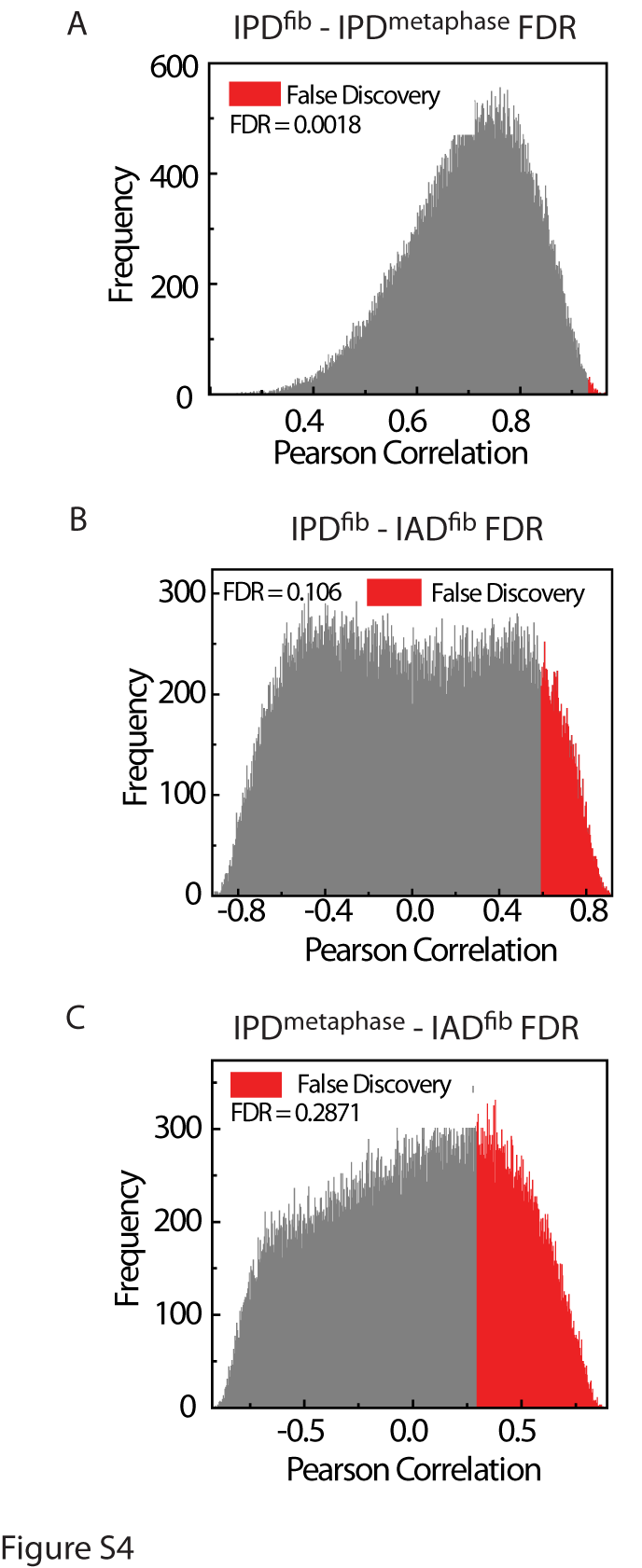

Supplement: Figure S4 — False Discovery rate estimation. (A) Histogram of PCC values for correlation between IPDfib and IPDprometaphase for 105 different randomized IPDrand matrices. Gray bars indicate PCC values less than the PCC value of unrandomized matrix (PCC0) and red indicates the values greater than PCC0. False discovery rate (FDR) is computed as the fraction of PCC values above PCC0. Histogram similar to (A) are shown for correlation between IPDfib and IADfib (B) and for correlation between IPDprometaphase and IADfib (C). (TIF) [file pone.0046628.s004.tif]

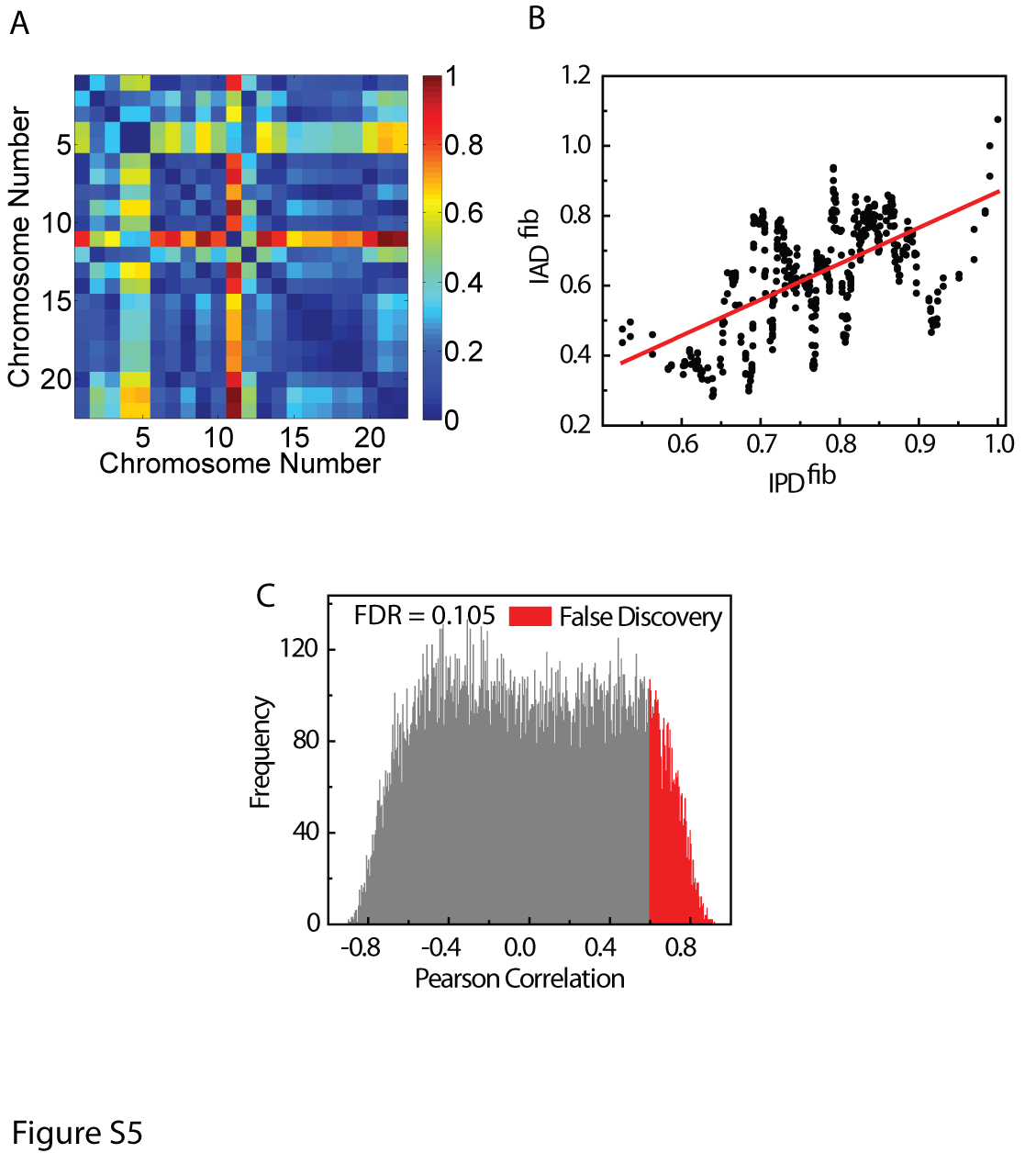

Supplement: Figure S5 — Correlation between IPD of fibroblast and IAD of fibroblast from selected genes. (A) IADselect of fibroblast generated from stringently selected genes with expression higher than 0.4 times the mean expression of the genes in a chromosome. (B) Correlation between IPDfib and IADselect. (C) Distribution of PCC for correlation between 30,000 different randomized IPD matrices and IADselect. The grey bars indicate PCCPCC0 (TIF) [file pone.0046628.s005.tif]

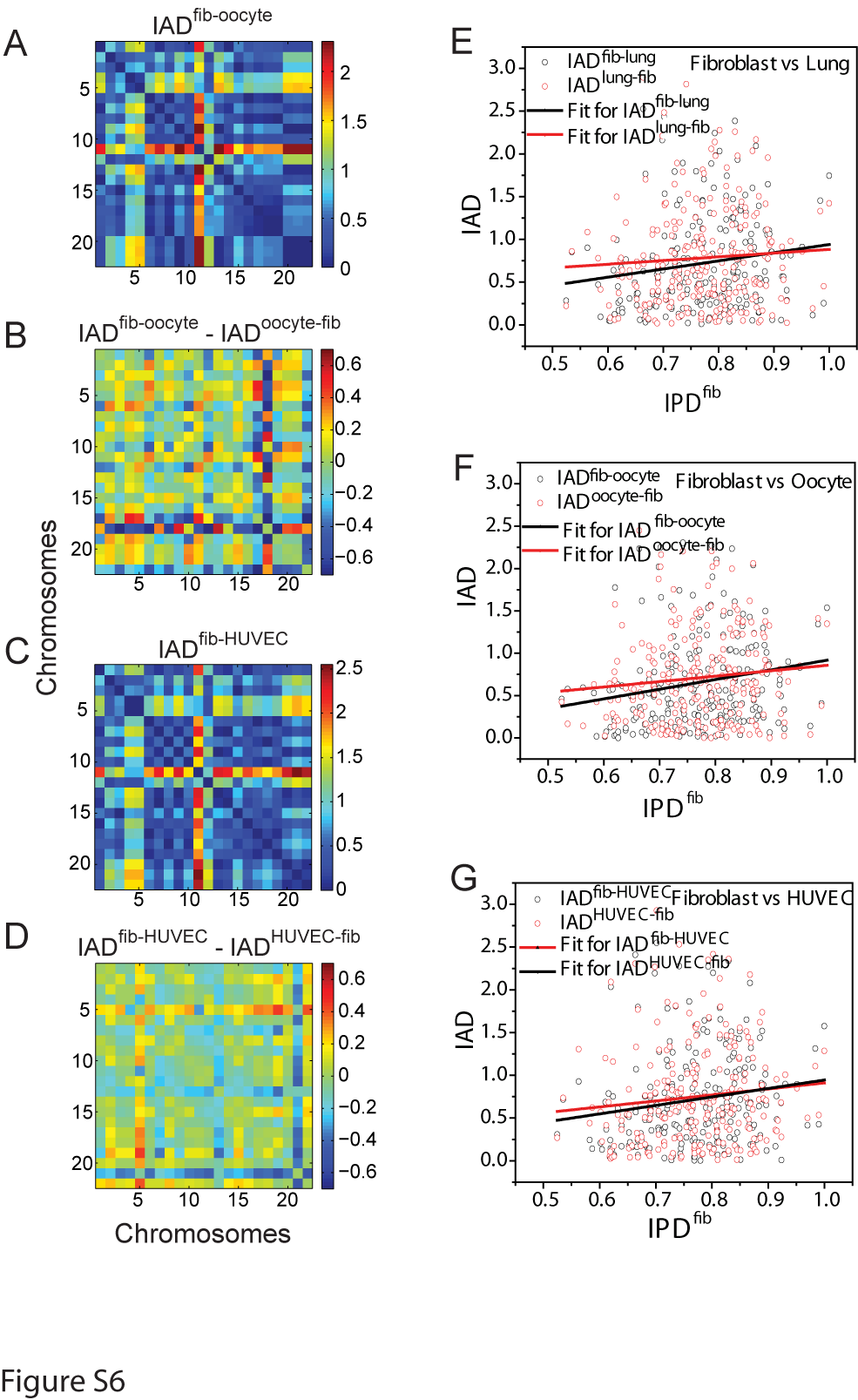

Supplement: Figure S6 — Matrices and scatter plot for comparison of fibroblast with other cell types. (A) IADfib-oocyte matrix is generated from the activity in fibroblast for differentially expressing gene between fibroblast and oocyte. (B) Difference between IADfib-oocyte and IADoocyte-fib, where IADoocyte-fib is the matrix generated from activity in oocyte for differentially expressing gene between fibroblast and oocyte. (C) and (D) are computed similarly as matrices in (A) and (B) respectively for IADfib-HUVEC. (E-G) Scatter plot and corresponding fits between IPDfib and IADfib-other or IADother-fib where in (E) other = lung, (F) other = oocyte and (G) other = HUVEC. (TIF) [file pone.0046628.s006.tif]

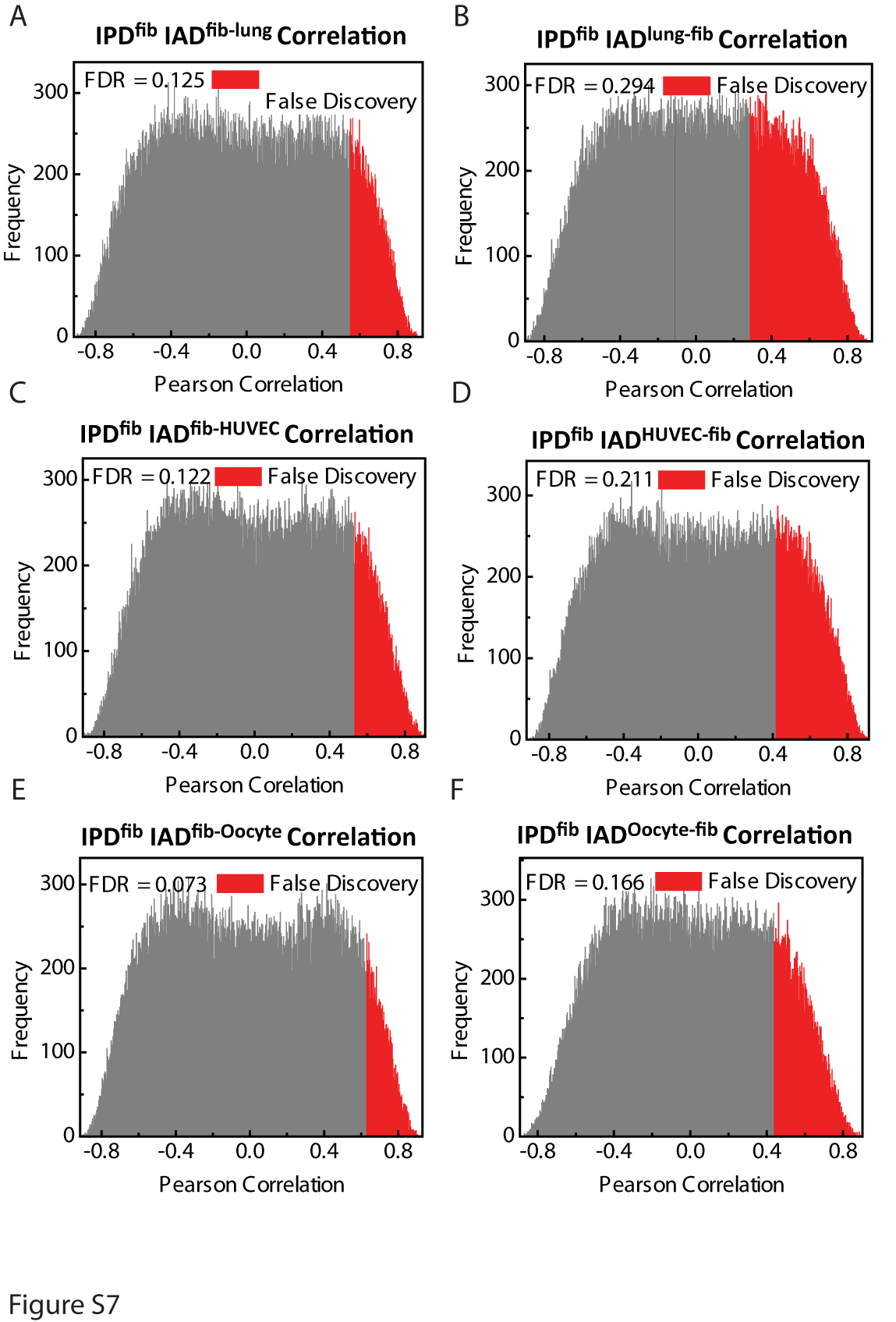

Supplement: Figure S7 — Estimation of false discovery rates for IPD and IAD correlations for different cell types. (A–F) Grey lines indicate PCC values less than the PCC value of the unrandomized matrix (PCC0), whereas red lines indicate PCC values greater than PCC0. FDR is computed as a fraction of the PCC values above PCC0. PCC for correlation between IPDfib and IADfib-other has a significantly smaller false discovery rates (FDR) as compared to the PCC value for correlation between IPDfib and IADother-fib. (TIF) [file pone.0046628.s007.tif]

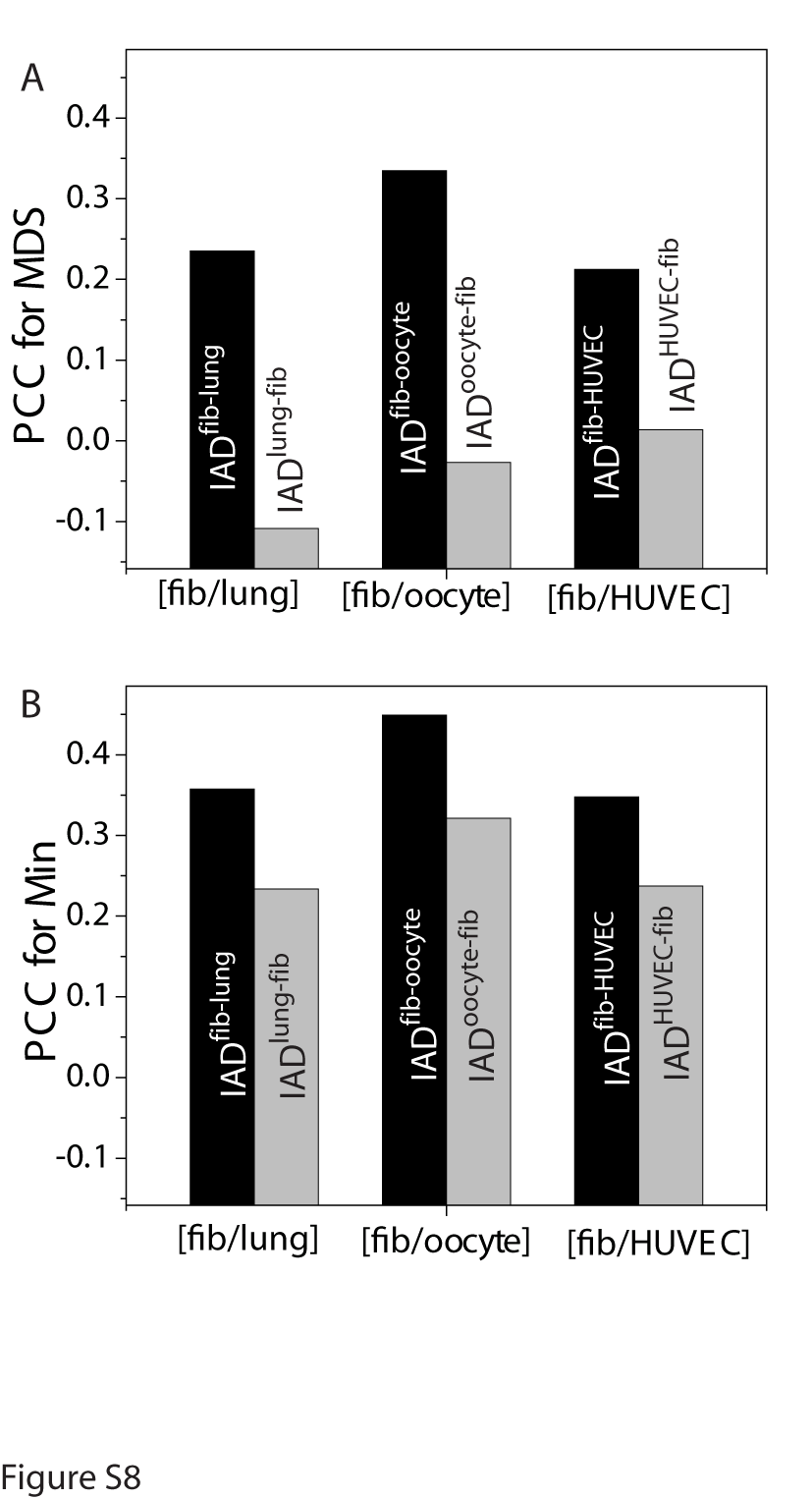

Supplement: Figure S8 — Pearson correlation and slope for the correlation between IPDfib and IADfib-other or IADother-fib generated from (A) minimum distances (IPDmin) and (B) MDS distances (IPDMDS) provided in Bolzer et al. (TIF) [file pone.0046628.s008.tif]

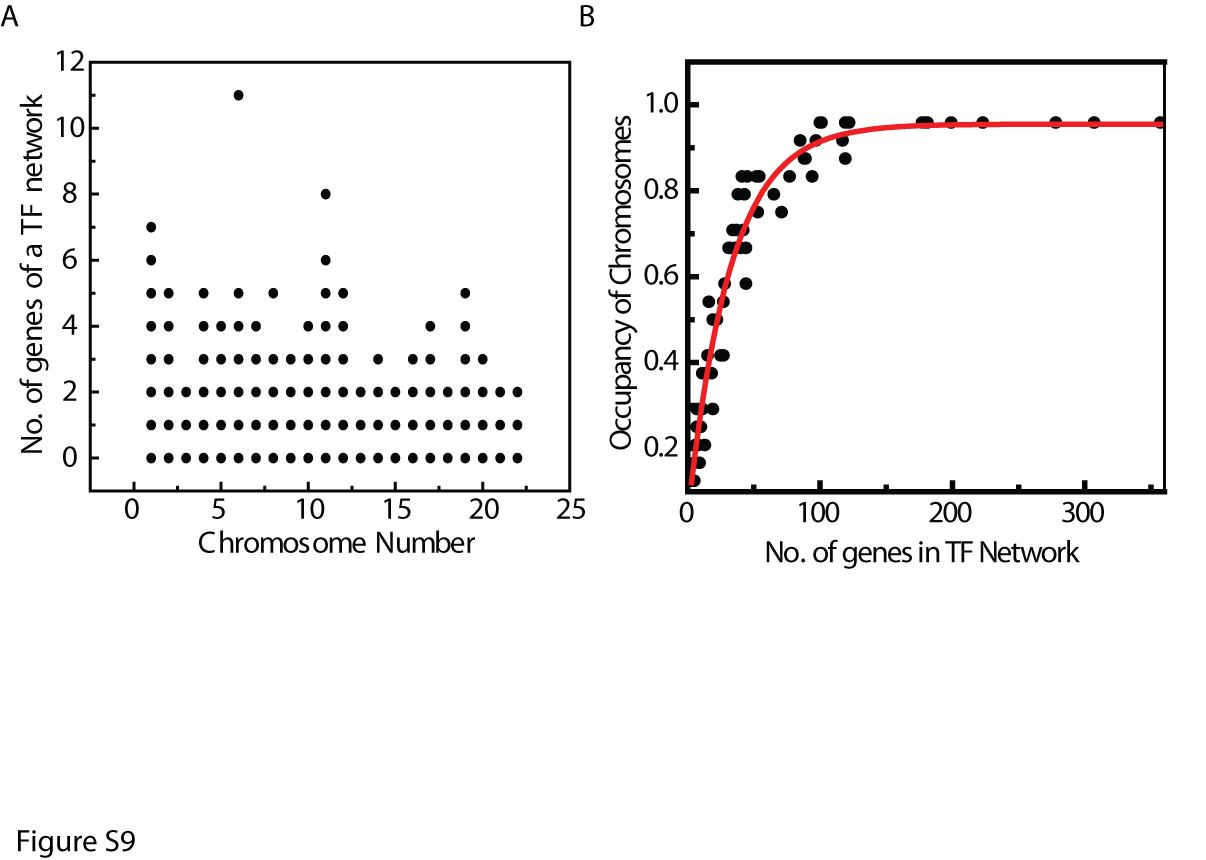

Supplement: Figure S9 — Characterization of TF networks. (A) Distribution of genes of a TF network (with<50 genes) over different chromosomes. The distribution of genes are not biased by size of the chromosome. (B) Occupancy of chromosomes for different TF networks. Occupancy is defined as the fraction of chromosomes having at least one gene from a TF network. (TIF) [file pone.0046628.s009.tif]

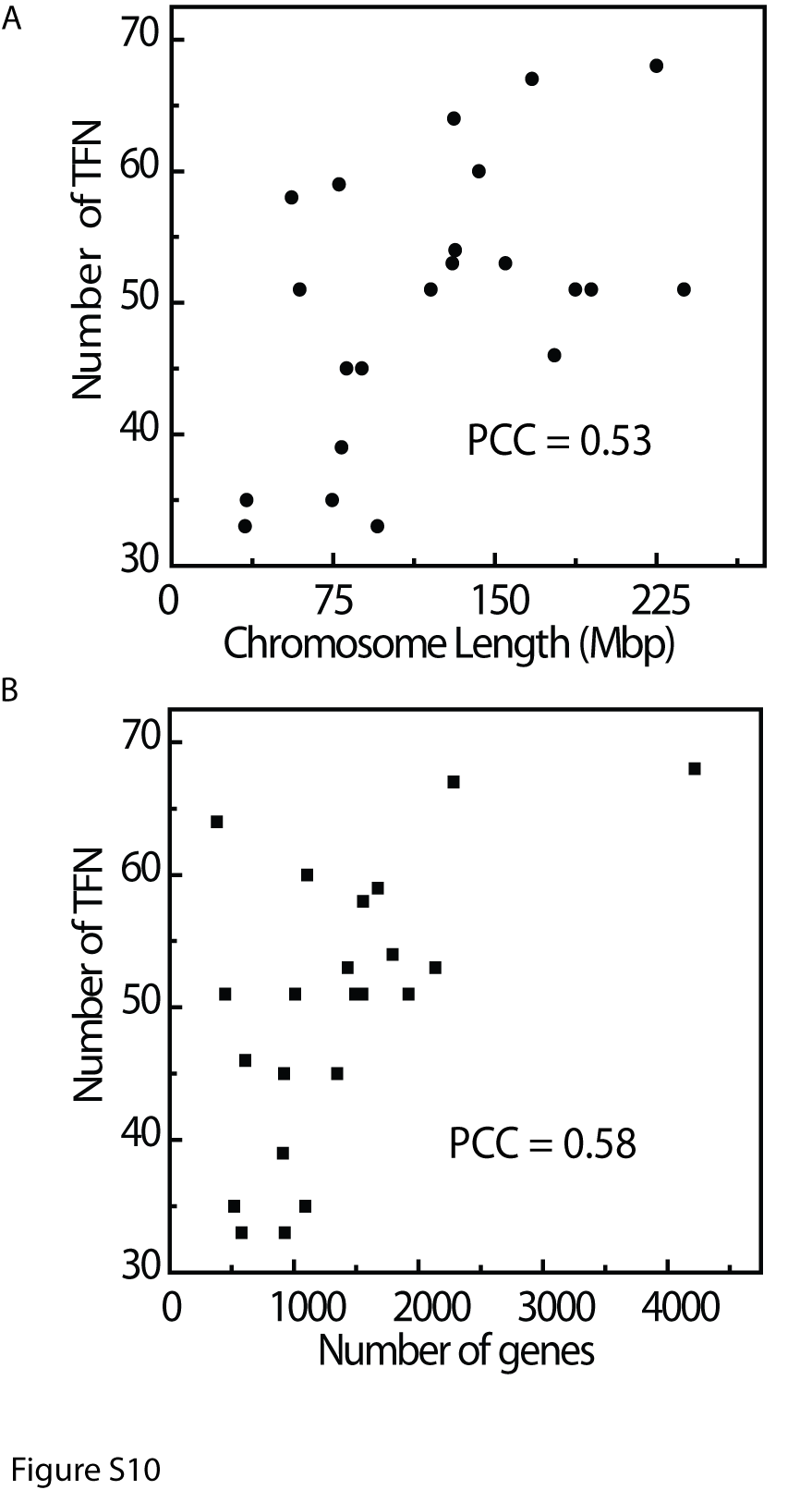

Supplement: Figure S10 — Dependance of Chromosomal association of TFs on chromosome length and number of genes. (A) Correlation between numbers of TFNs associated with a chromosome and the length of chromosome in base pairs, with a PCC of 0.53. (B) Correlation between number of TFNs associated with a chromosome and the number of annotated genes on that chromosome (PCC = 0.58). (TIF) [file pone.0046628.s010.tif]

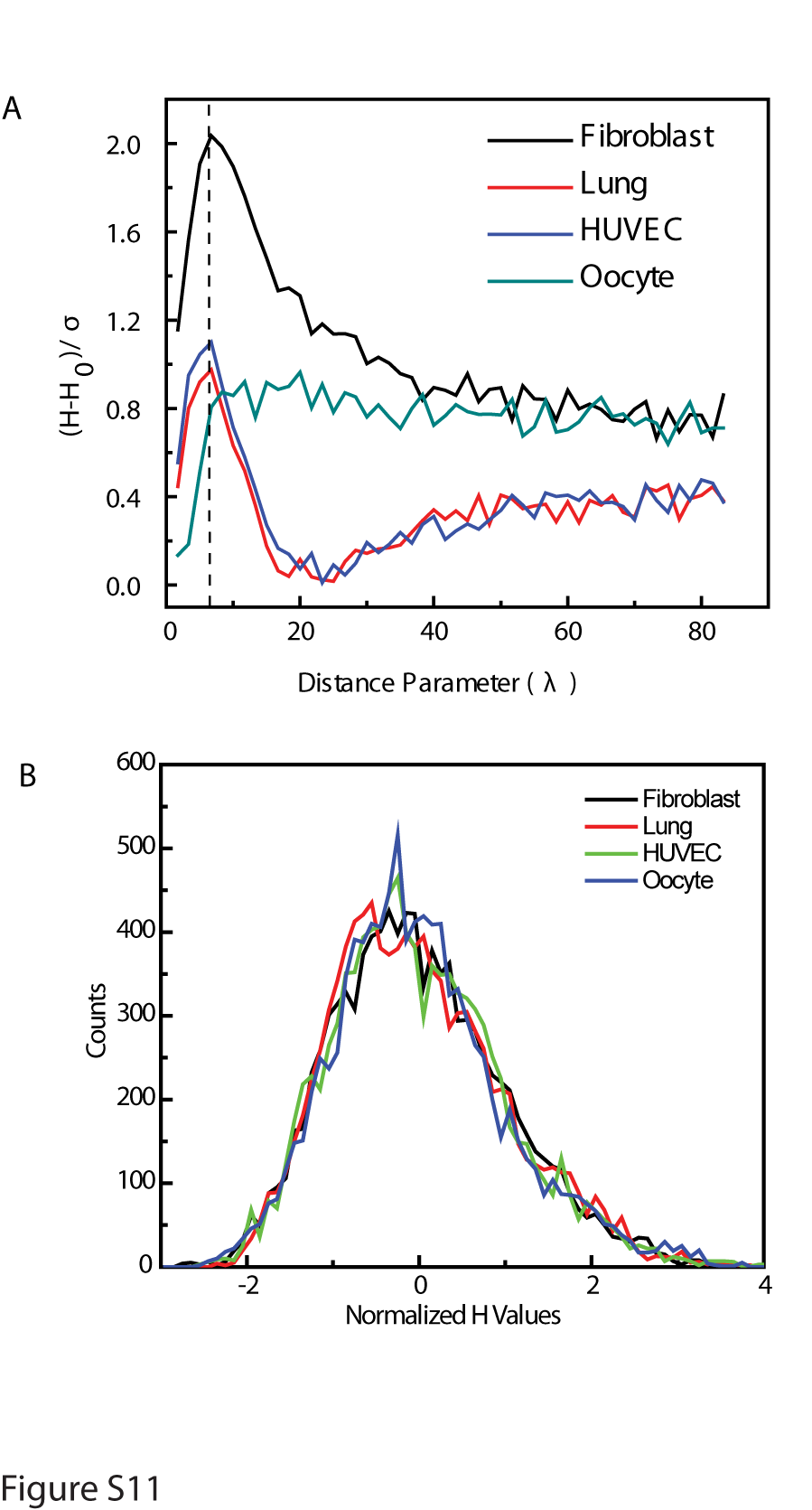

Supplement: Figure S11 — Dependence of H-values on the distance parameter. The variation of (H−H0)/σ with variation in the value of λ (in units of % nuclear radius), shows a maximum value at λ = 7% of nuclear radius for all the different cell types. (B) Histogram of H values for 10,000 iterations of randomization, computed for four different cell types. (TIF) [file pone.0046628.s011.tif]

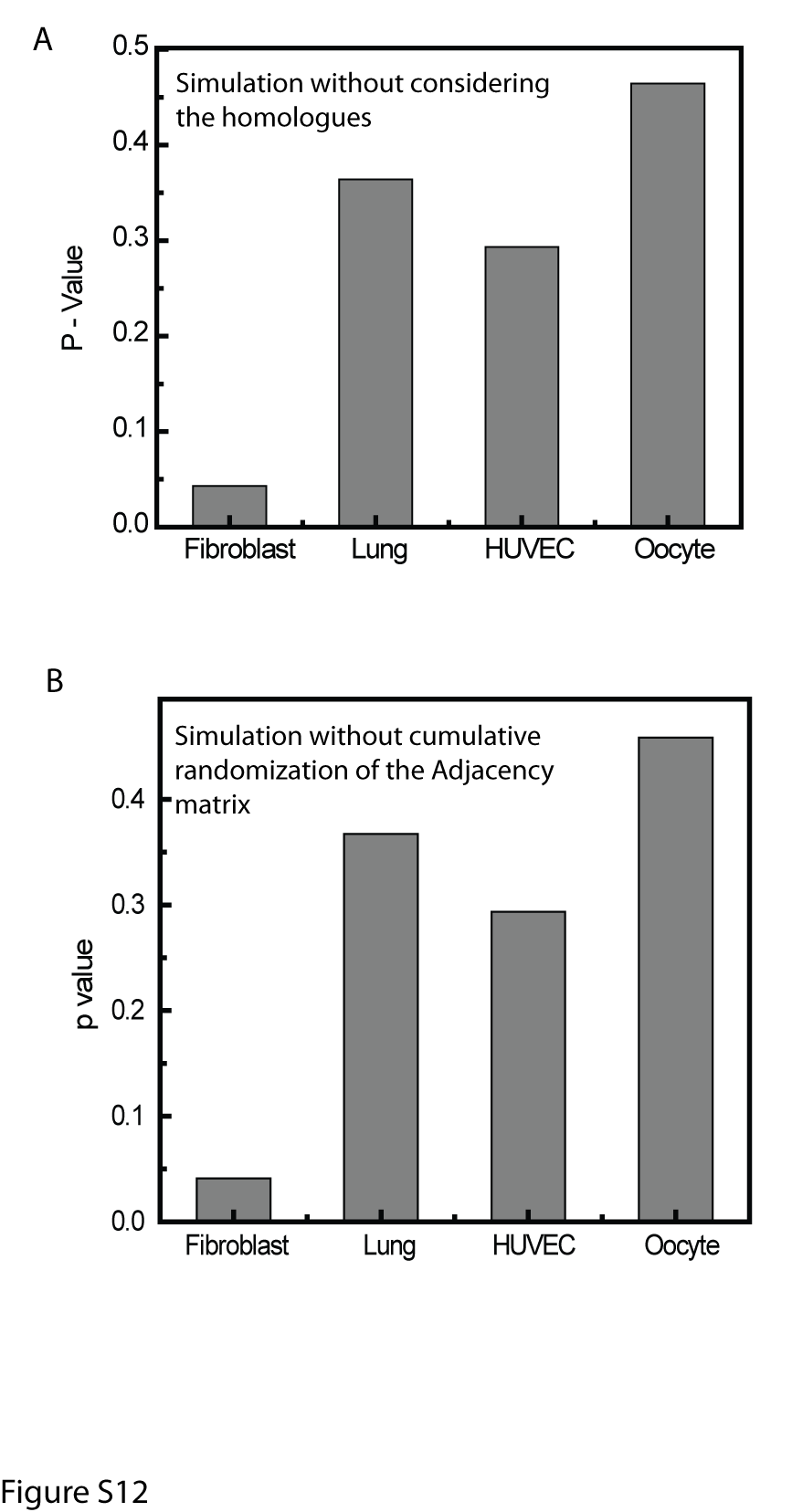

Supplement: Figure S12 — Different modes of simulation yield similar p values for different cell types. (A) p values obtained for estimation of H values without considering the adjacency matrix values for homologues. (B) p values obtained for estimation of H values without cumulative randomization of the adjacency matrix. H values in this case are computed after 100 steps of randomization of the adjacency matrix, for 10,000 iterations. (TIF) [file pone.0046628.s012.tif]

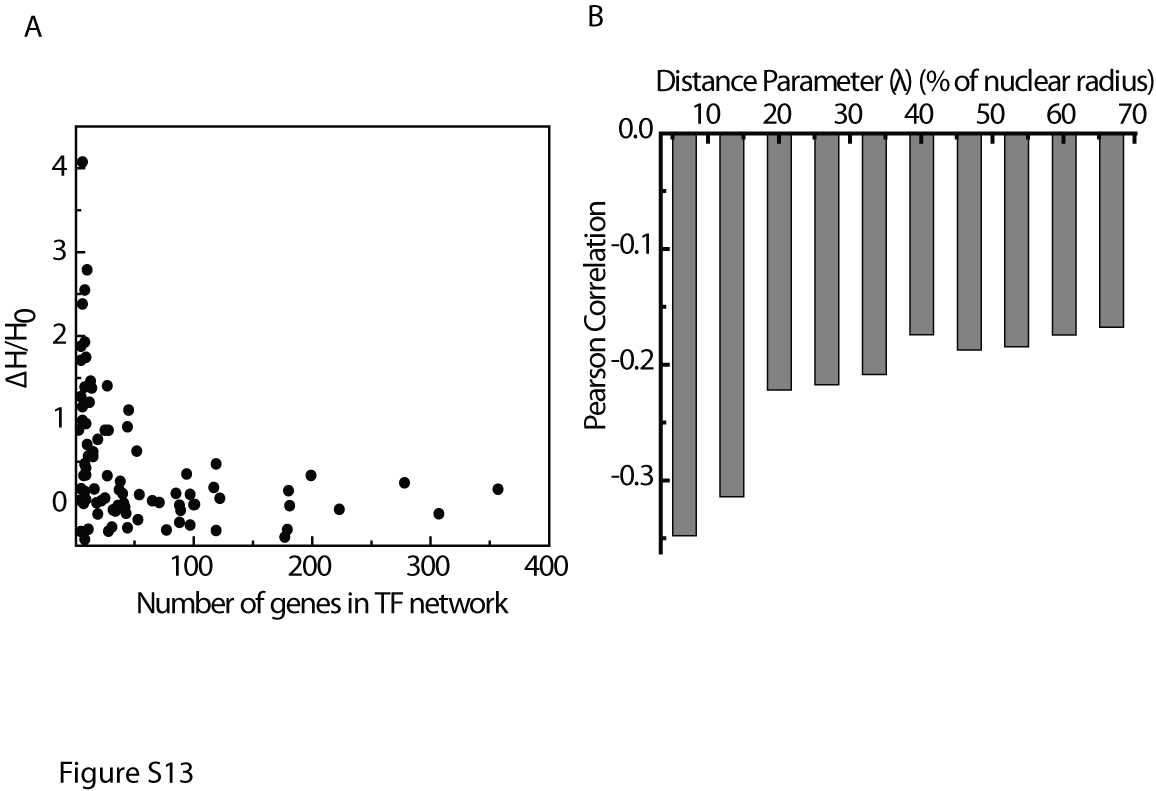

Supplement: Figure S13 — Dependance of Change in H value upon the number of genes in a TF network. (A) Correlation between ΔH/H0 and number of genes in a TF network. (B) Pearson correlation coefficient of the correlation between ΔH/H0 and number of TF network depends on the distance parameter (λ). (TIF) [file pone.0046628.s013.tif]

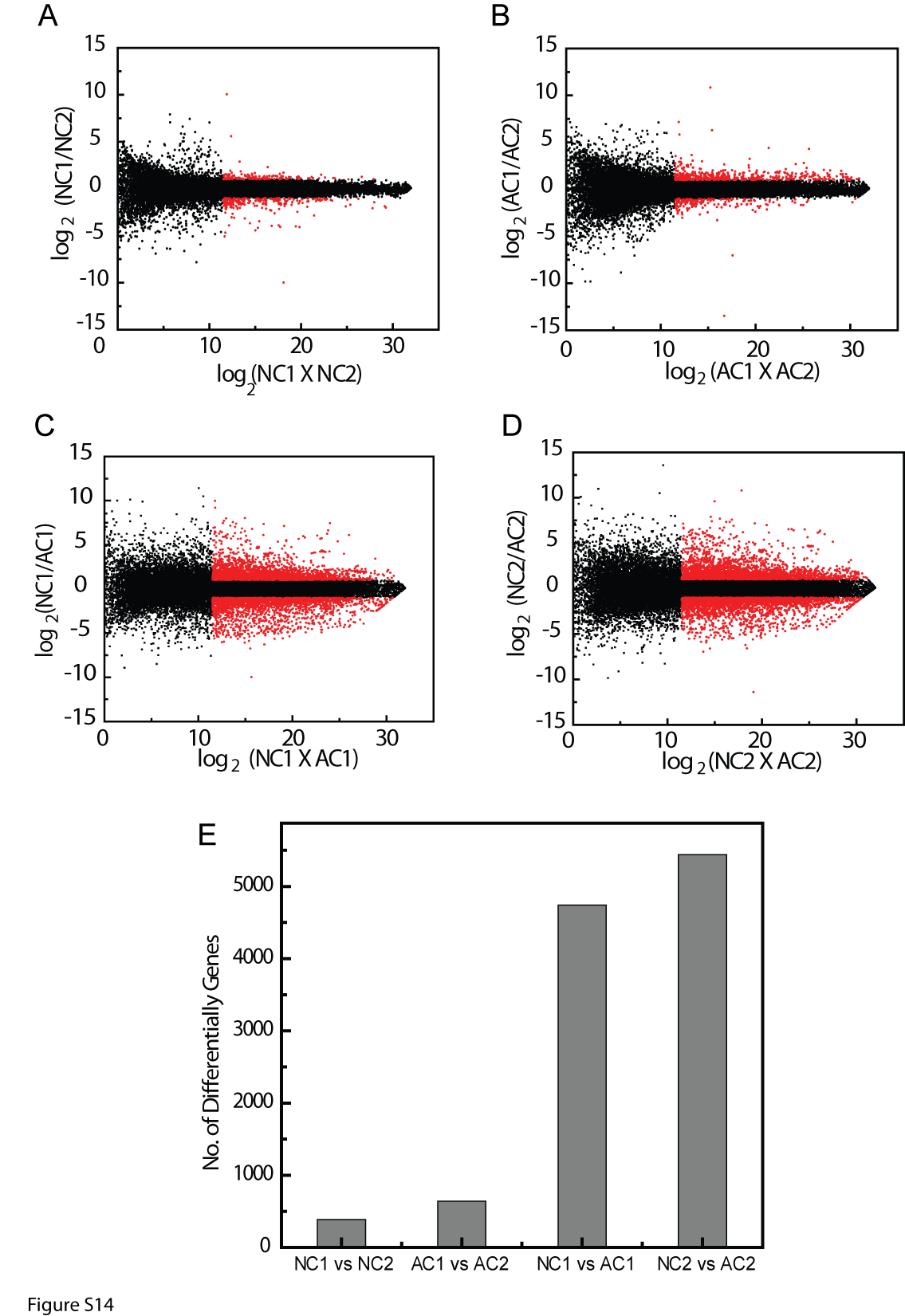

Supplement: Figure S14 — Similarity in gene expression across batches of T- cells. (A) scatter plot between log2 ratios of NC1 (Naïve T Cell, replicate 1) & NC2 (Naïve T cell, replicate 2) , and log2 intensity of NC1 and NC2, showing that there are very few differentially expressing genes across two different batches of Naïve T cells. (B) Scatter plot similar to (A) between AC1(Activated T cell, replicate 1) and AC2 (Activated T cell, replicate 2). (C) Scatter plot between log2 ratios of NC1 and AC1, and log2 intensities of NC1 and AC1, showing large number of diiferentially expressing genes, when Naïve and Activated T cells of the same batch are considered. (D) Scatter plot similar to (C) between NC2 and AC2. (E) Number of differentially expressing genes when either naïve or activated T cells of different batches are considered (NC1 vs NC2 and AC1 vs AC2), or when naïve and activated T cells of the same batch are compared (Nc1 vs AC1 and NC2 vs AC2). (TIF) [file pone.0046628.s014.tif]

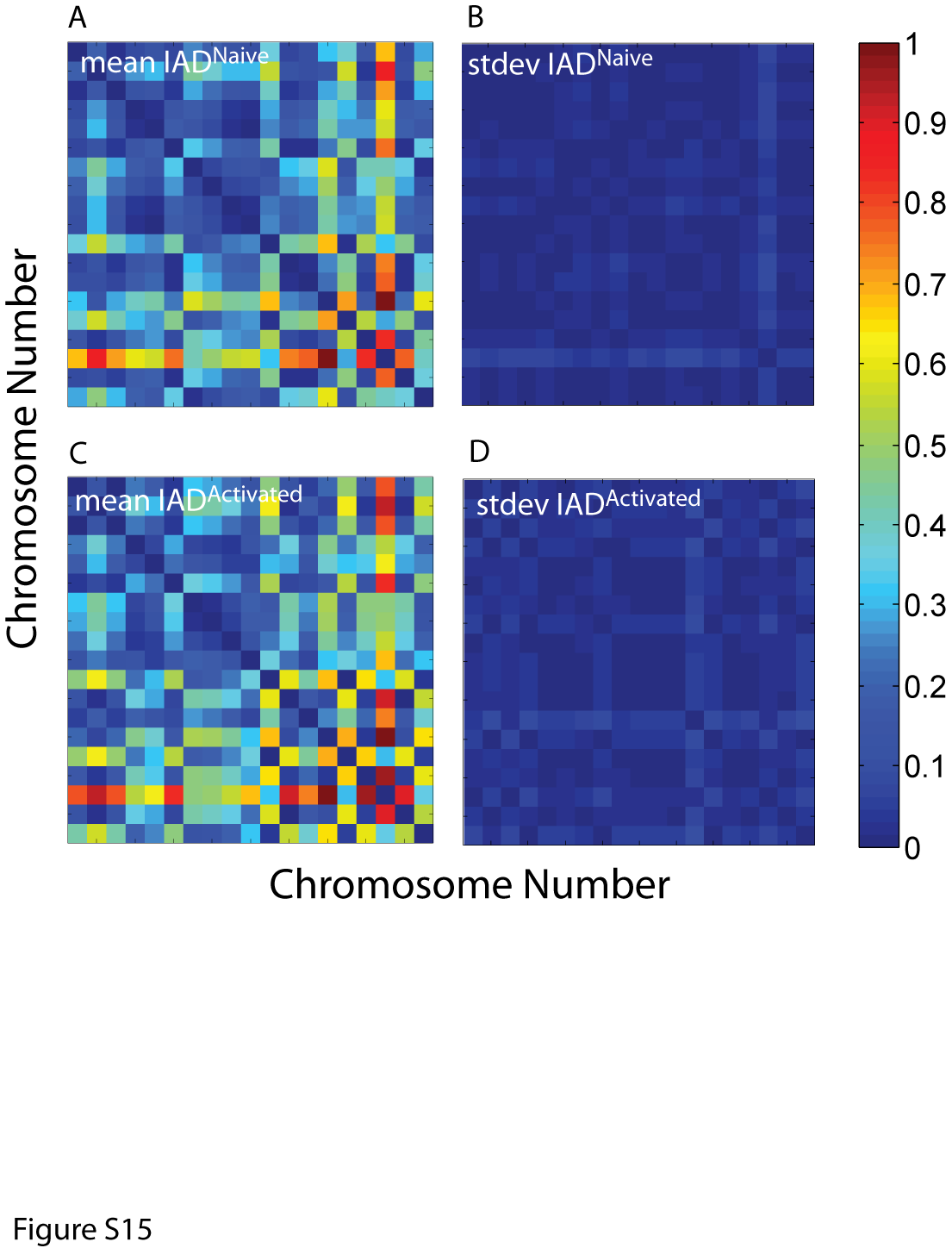

Supplement: Figure S15 — Variability in IAD matrix across batches of cells. (A) Mean IADNaive matrix averaged over two replicates of microarray from two batches of cells. (B) Matrix showing the standard deviation in the value of IADNaive matrix. (C) Mean IADActivated matrix. (D) Standard deviation in estimation of IADActivated. (TIF) [file pone.0046628.s015.tif]

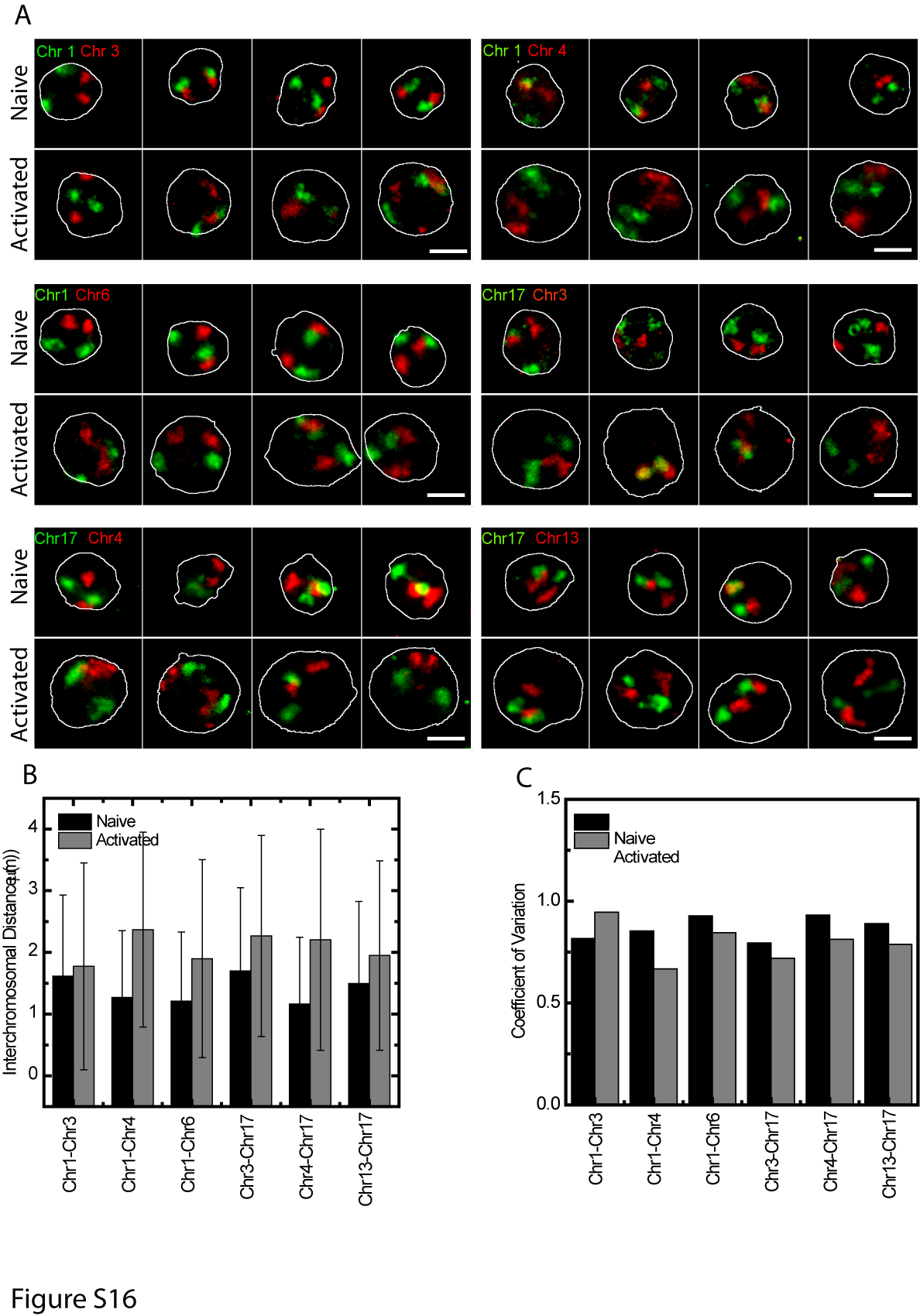

Supplement: Figure S16 — 3D Chromosome FISH in Naïve and Activated T-Cells. (A) Images of nuclei showing 6 different chromosome pairs labeled in mouse naïve and activated T-cells. White outline indicates the boundary of the nucleus. (B) Interchromosomal Interface distances in Naïve and activated T cells. (B) Coefficient of variation (σ/µ) computed for the interchromosome interface distances for naïve and activated T cells. Scale bar, 5 µm. (TIF) [file pone.0046628.s016.tif]

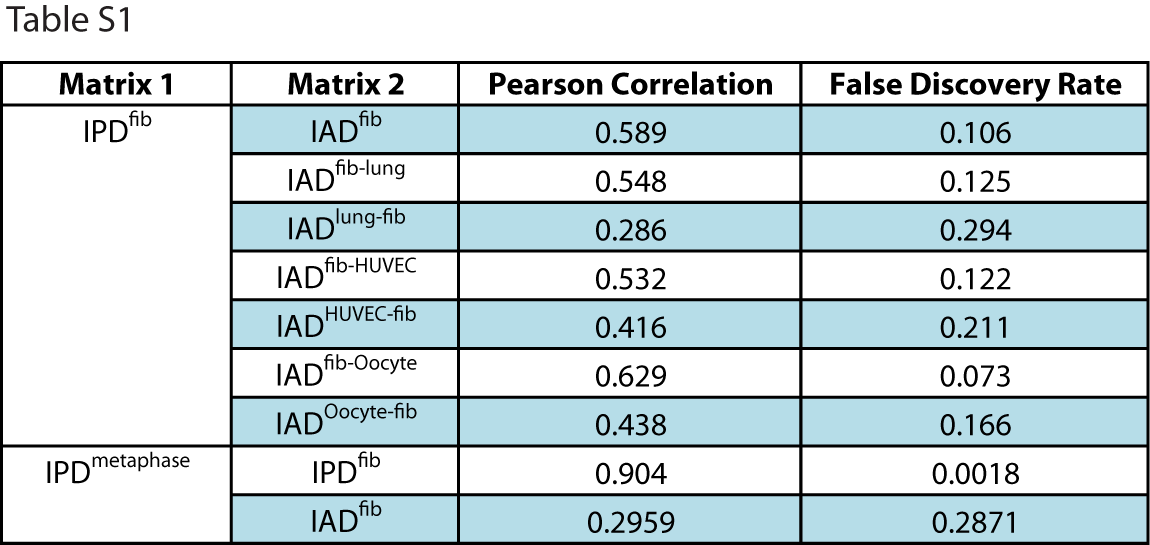

Supplement: Table S1 — Pearson Correlation and FDR Table. The table shows the Pearson correlation and the corresponding false discovery rates when Matrix 1 is correlated with Matrix 2 (TIF) [file pone.0046628.s017.tif]

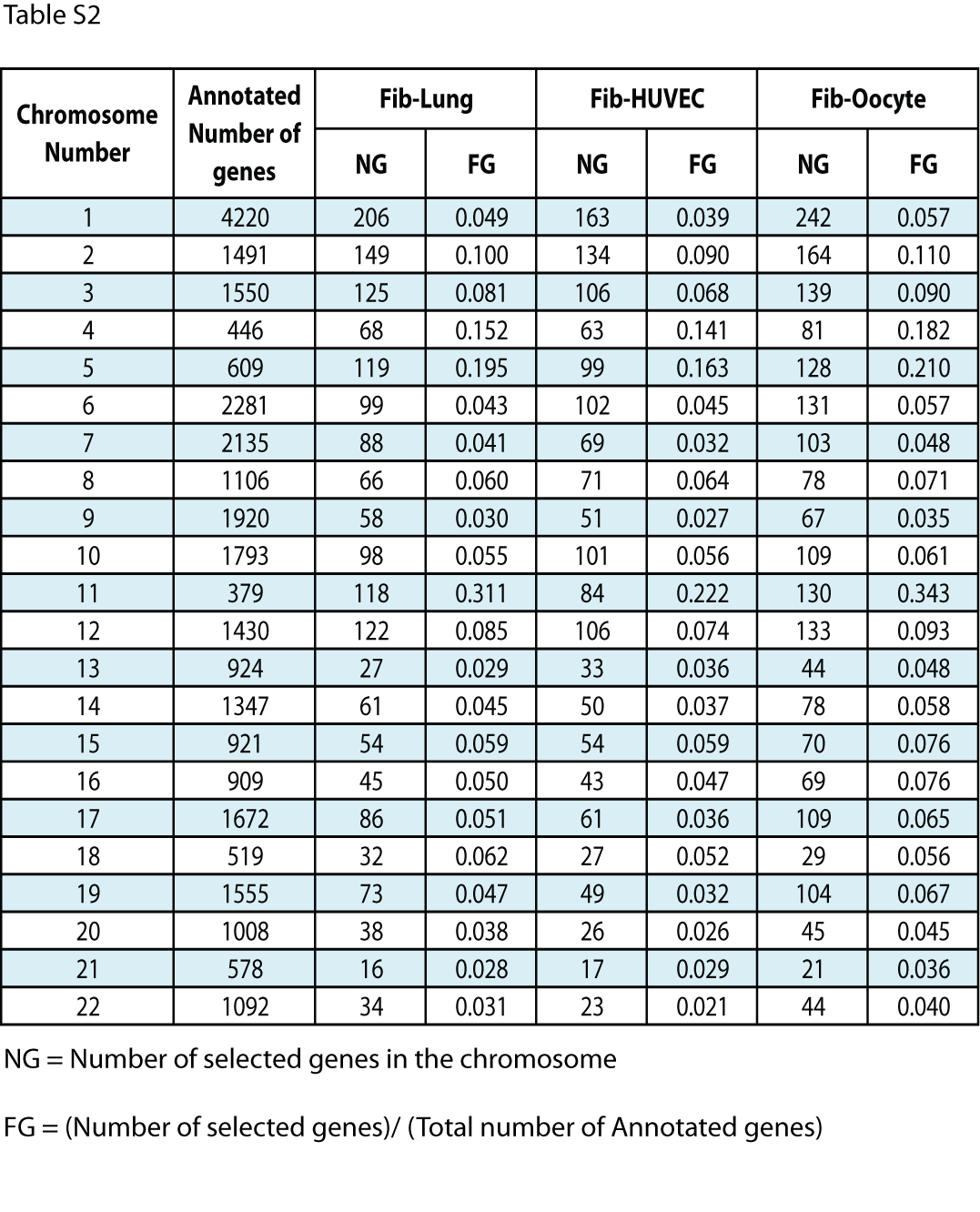

Supplement: Table S2 — Table of Differentially expressed genes within cell types. The table represents the total number of annotated genes and the number of genes selected for differential expression between fibroblast and other cell types. (TIF) [file pone.0046628.s018.tif]

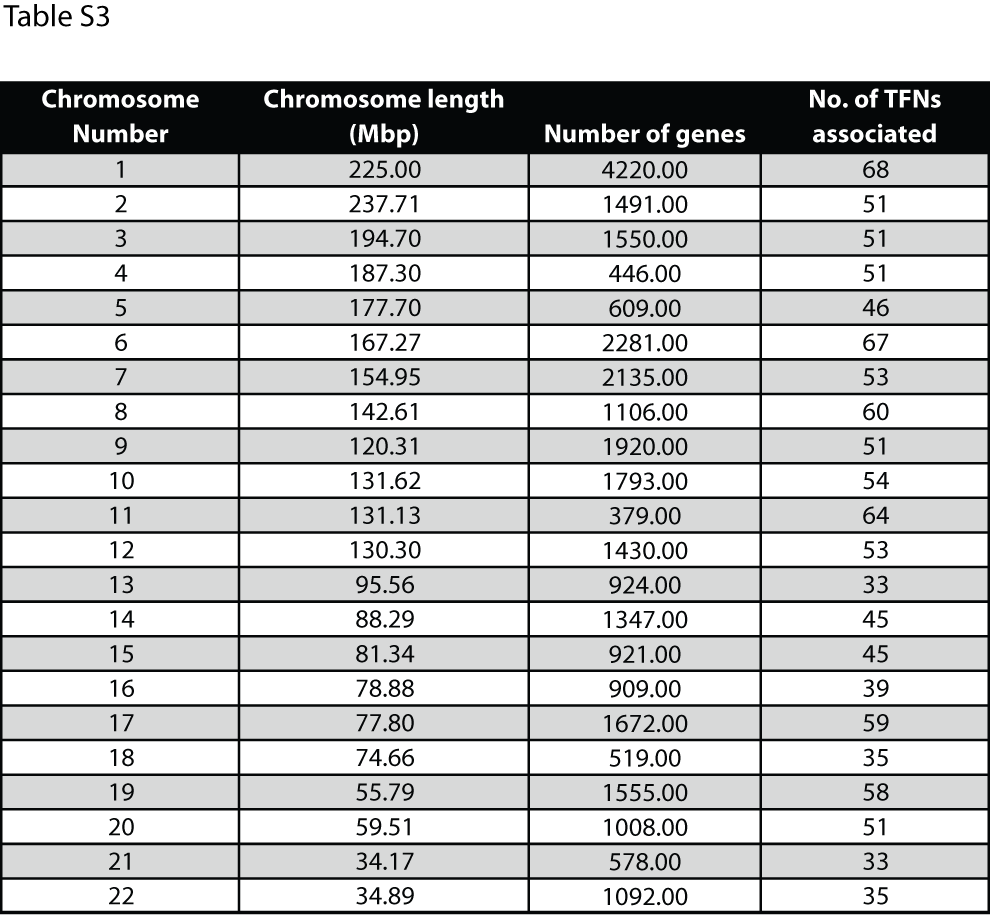

Supplement: Table S3 — Transcription Factor Network Association with chromosomes. This table provides the number of Transcription Factor networks associated with different chromosomes. (TIF) [file pone.0046628.s019.tif]

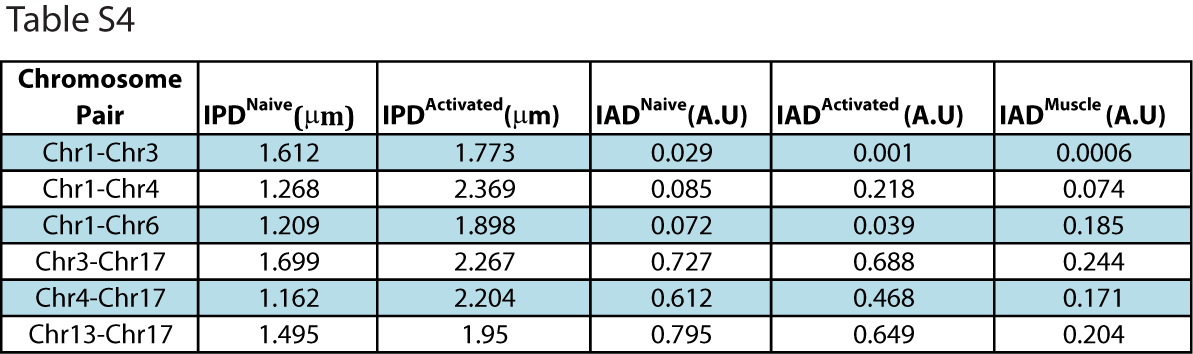

Supplement: Table S4 — T-Cell IPD and IAD table. The table shows the IPD for naïve and activated T-Cell for selected pairs of chromosomes and IAD for Naïve, Activated and Muscle cells for the same pair of chromosomes. (TIF) [file pone.0046628.s020.tif]

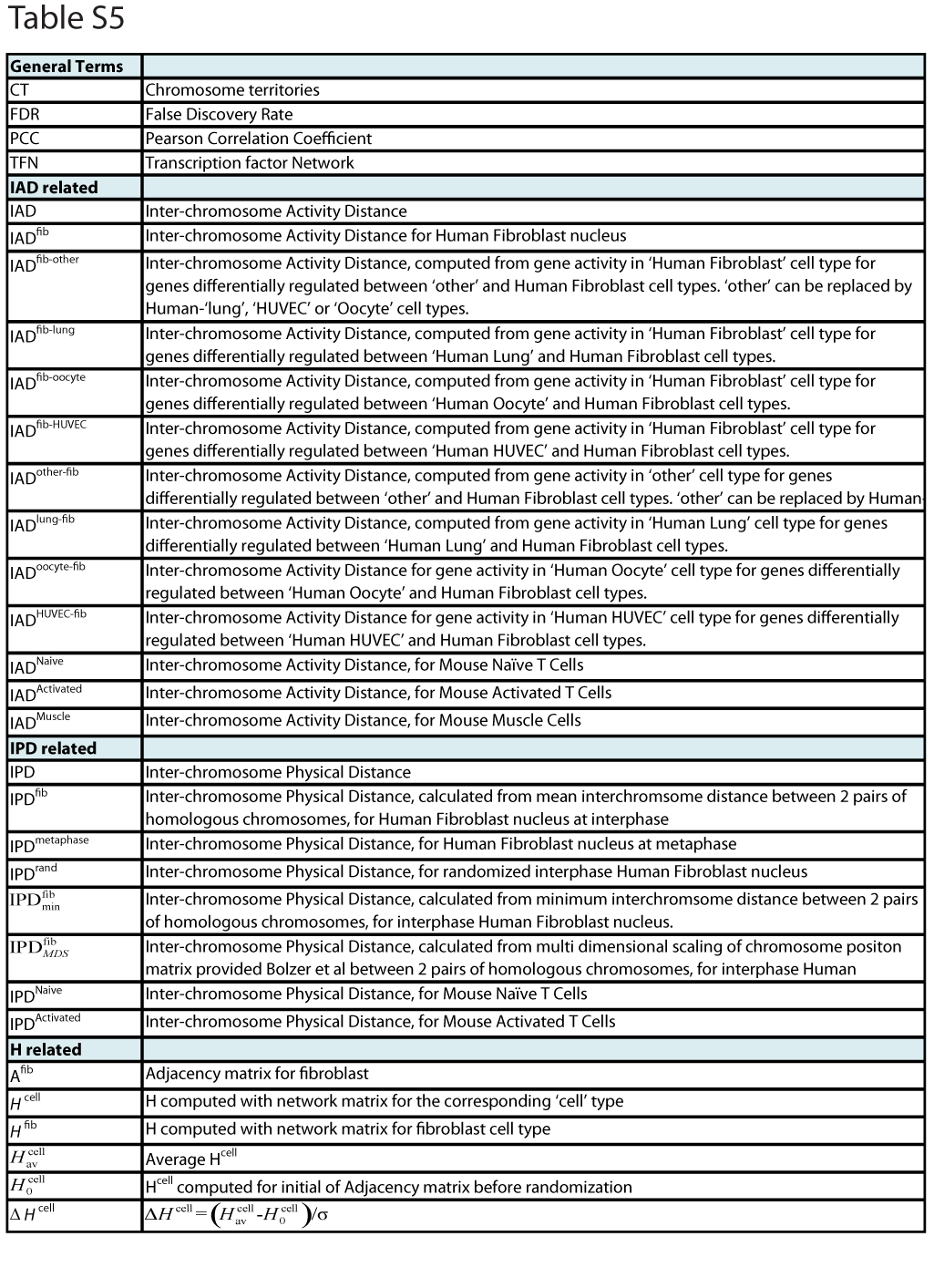

Supplement: Table S5 — Table of Abbreviations. This table provides the meaning of the abbreviations and notations used in the manuscript. (TIF) [file pone.0046628.s021.tif]
